# Supramolecular Switch for the Regulation of Antibacterial Efficacy of Near-Infrared Photosensitizer

**DOI:** 10.3390/molecules29051040

**Published:** 2024-02-28

**Authors:** Yu-Na Jiang, Manqi Tan, Chenglong He, Jiaxi Wang, Yi Wei, Ningning Jing, Bing Wang, Fang Yang, Yujie Zhang, Meng Li

**Affiliations:** 1 Cixi Biomedical Research Institute, Wenzhou Medical University, Ningbo 315302, China; jiangyuna@nimte.ac.cn (Y.-N.J.); tanmanqi@nimte.ac.cn (M.T.); hechenglong@nimte.ac.cn (C.H.); 2 Ningbo Key Laboratory of Biomedical Imaging Probe Materials and Technology, Ningbo Institute of Materials Technology and Engineering, Chinese Academy of Sciences, Ningbo 315201, China; wangbing@nimte.ac.cn; 3 School of Materials Science and Engineering, University of Science and Technology Beijing, Beijing 100083, China; jiaxi0726@163.com (J.W.); weiyi5166@126.com (Y.W.); 4 College of Science and Technology, Ningbo University, Ningbo 315300, China; jingnn7009@163.com; 5 Zhejiang International Scientific and Technological Cooperative Base of Biomedical Materials and Technology, Ningbo Cixi Institute of Biomedical Engineering, Ningbo 315300, China

**Keywords:** near-infrared photosensitizers, photodynamic antimicrobials, host-guest interactions, nanoassemblies, supramolecular complexes

## Abstract

The global antibiotic resistance crisis has drawn attention to the development of treatment methods less prone to inducing drug resistance, such as antimicrobial photodynamic therapy (aPDT). However, there is an increasing demand for new photosensitizers capable of efficiently absorbing in the near-infrared (NIR) region, enabling antibacterial treatment in deeper sites. Additionally, advanced strategies need to be developed to avert drug resistance stemming from prolonged exposure. Herein, we have designed a conjugated oligoelectrolyte, namely TTQAd, with a donor-acceptor-donor (D-A-D) backbone, enabling the generation of reactive oxygen species (ROS) under NIR light irradiation, and cationic adamantaneammonium groups on the side chains, enabling the host-guest interaction with curcubit[7]uril (CB7). Due to the amphiphilic nature of TTQAd, it could spontaneously form nanoassemblies in aqueous solution. Upon CB7 treatment, the positive charge of the cationic adamantaneammonium group was largely shielded by CB7, leading to a further aggregation of the nanoassemblies and a reduced antibacterial efficacy of TTQAd. Subsequent treatment with competitor guests enables the release of TTQAd and restores its antibacterial effect. The reversible supramolecular switch for regulating the antibacterial effect offers the potential for the controlled release of active photosensitizers, thereby showing promise in preventing the emergence of drug-resistant bacteria.

## 1. Introduction

Bacterial infection is a serious threat to human life and health [1]. The use of antibiotics has successfully reduced the morbidity and mortality of bacterial infections to a certain extent. However, the emergence of drug-resistant pathogen infections has precipitated a global public health crisis due to antibiotic abuse and misuse [2]. Therefore, it is crucial to develop new methods to slow down or prevent the emergence of drug-resistant bacteria for effective bacterial infection treatment [3,4,5]. Antimicrobial photodynamic therapy (aPDT) represents an innovative therapeutic modality, employing specifically designed photosensitizers (PSs) in conjunction with an excitation light source to facilitate the generation of reactive oxygen species (ROS) [6]. These ROS are equipped with the capacity to induce oxidative damage to an array of biomolecules such as lipids, proteins, and nucleic acids, thereby leading to the eradication of tumor cells or pathogenic microorganisms [7,8]. APDT is a clinical treatment method that has attracted tremendous attention in recent decades because of its temporal and spatial selectivity and broad antimicrobial spectrum, and it is less prone to inducing drug resistance [9,10]. However, the clinical application of aPDT is limited because most reported photosensitizers require excitation under ultraviolet or short-wavelength visible light, which inevitably leads to cellular phototoxicity and limited tissue penetration depth [11,12]. Recent investigations have elucidated that near-infrared (NIR) light demonstrates exceptional tissue penetration capabilities and concurrently minimizes collateral damage to normal cells, thereby offering significant benefits and propelling the advancement of NIR light-excited photosensitizers [13].

Conjugated oligoelectrolytes (COE) are a class of organic small molecules with conjugated backbones and charged side chains [14,15]. The introduction of electron donor-acceptor (D-A) structures to the conjugated backbone endows the COE with long-wavelength absorption owing to the efficient intramolecular charge transfer (ICT) from electron-rich to electron-deficient moieties [16]. In addition, the D-A structure in the backbone also promotes the intersystem crossing (ISC) process upon the photoexcitation of the COE, thus favoring the following photosensitizing process in the triplet state [17,18]. Furthermore, the binding capacity of COE against pathogenic microorganisms can be adjusted by strategically engineering their side chains. For instance, incorporating cationic side chains in COE enhances their binding affinity to bacteria with negatively charged membranes [19,20]. Therefore, a number of COE have been reported as potential photosensitizers in the photodynamic antibacterial treatments [21,22,23,24]. However, with the ability to evolve and adapt, the bacteria may still manifest resistance to aPDT under prolonged exposure to photosensitizers [25,26]. Therefore, there is a need to develop methods with an on-demand exposure of photosensitizers to bacteria.

Supramolecular chemistry focuses on molecular recognition and highly organized molecular assemblies formed through non-covalent interactions [27]. These interactions include hydrogen bonds, hydrophobic interactions, electrostatic interactions, van der Waals forces, and π-π stacking. Because of the dynamic nature of these non-covalent interactions, the supramolecular assemblies are often stimuli-responsive, making them highly promising for biomedical applications [28]. Cucurbit[n]uril (CBn) is a class of synthetic host molecules characterized by a distinctive pumpkin-shaped structure. Their hydrophobic cavities and multicarbonyl structures at the portals facilitate the formation of stable host-guest supramolecular complexes with various guest molecules, such as cationic antibacterial agents, through hydrophobic, electrostatic, and dipole-dipole interactions. Thus, CBn can be utilized to regulate the antibacterial activity of its host molecule in a stimuli-responsive and reversible manner [29,30,31]. In 2015, Wang’s team proposed a supramolecular strategy to reversibly turn-on and turn-off of the photodynamic antibacterial activity of a cationic poly(phenylene vinylene) derivative (PPV) based on the host-guest assembly between PPV and CB7 [32]. Cationic PPV can form a non-covalent supramolecular complex with CB7, thus reducing the photodynamic antibacterial activity of PPV. The addition of competitive molecules (amantadine, AD) forms a more stable CB7/AD complex, effectively restoring the photodynamic antibacterial activity of PPV. In 2022, Qi’s group constructed a hemicyanine supramolecular assembly between hemicyanines and CB7, and also achieved a reversible “switching off” or “switching on” of hemicyanine’s photodynamic antibacterial activity through supramolecular self-assembly or disassembly [33]. Supramolecular encapsulation by CB7 has been demonstrated as a straightforward and efficacious strategy for the modulation of antibacterial activity in antibiotics [34,35]. Similarly, Wang’s team has successfully constructed a supramolecular “antibiotic switch” for a facile “plug-and-play” regulation of antibacterial efficacy via the supramolecular self-assembly and disassembly of the three constituent components: berberine (BBR), CB7, and competitor AD [36]. However, the currently used photosensitizers are still white-light-activated. Additionally, due to the low binding constants of the reported photosensitizers with CB7, a high concentration of CB7 (over 10 equivalents) and competitive molecules (over 50 equivalents) are required in the above systems. Hence, there is a need for the development of photosensitizers with high binding affinities with CB7.

**Scheme 1:**
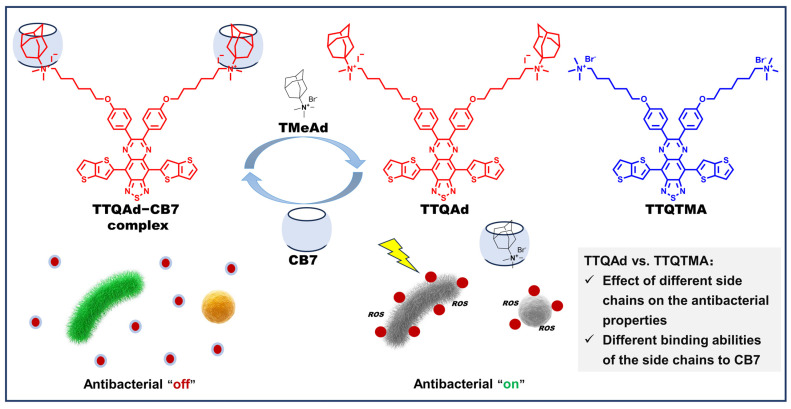
Chemical structures of TTQAd and TTQTMA, and schematic diagram of reversible regulation of antibacterial activity of TTQAd through supramolecular assembly or disassembly.

**Figure 1:**
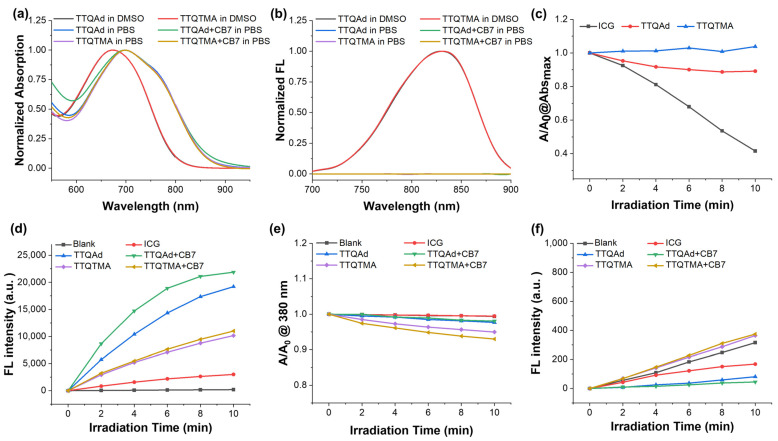
(**a**) Absorption spectra of TTQAd and TTQTMA in DMSO and PBS before and after the addition of CB7. (**b**) Fluorescence spectra of TTQAd and TTQTMA in DMSO and PBS before and after the addition of CB7. (**c**) Relative changes in the maximum absorbances of TTQAd, TTQTMA, and ICG in PBS irradiated by an 808 nm laser. (**d**) Changes in the fluorescence intensities of DCFH at 525 nm in the presence of TTQAd, TTQTMA, and ICG under the 808 nm laser before and after the addition of CB7. (**e**) Changes in the absorbances of ABDA at 380 nm in the presence of TTQAd, TTQTMA, and ICG under the 808 nm laser before and after the addition of CB7. (**f**) Changes in the fluorescence intensities of SOSG at 525 nm in the presence of TTQAd, TTQTMA, and ICG under the 808 nm laser before and after the addition of CB7.

Herein, we have designed a conjugated oligoelectrolyte TTQAd with thieno[3,2-b]thiophene as the electron donor (D) and thiadiazoloquinoxaline (TQ) as the electron acceptor (A) in the backbone for long-wavelength absorption, and cationic adamantaneammonium groups on the side chains for controlled interaction with bacteria (Figure 1). Benefiting from its D-A-D structure, TTQAd exhibits efficient absorption in the first near-infrared region (NIR-I) and the ability to generate ROS when subjected to 808 nm laser irradiation, enabling photodynamic antimicrobial activity in the NIR-I. Due to the ultrastable host-guest interaction between adamantaneammonium groups and CB7, the positive charge of TTQAd can be shielded by CB7 upon the formation of the TTQAd-CB7 supramolecular complex. Therefore, the antibacterial effect of TTQAd can be “turned off” after its complexation with CB7 and then “turned on” again upon treatment with competitor guests *N*,*N*,*N*-trimethyladamantan-1-aminium bromide (TMeAd) (Scheme 1), which allowed for the controlled release of the photosensitizer, preventing prolonged exposure to bacteria and thereby showing promise in slowing down the emergence of a drug-resistant strain.

## 2. Results and Discussion

### 2.1. Synthesis and Characterization of TTQAd and TTQTMA

The synthetic route of TTQAd and TTQTMA was shown in Scheme S1. The control molecule TTQTMA was designed and synthesized to investigate the effect of different side chains on the antibacterial properties and different binding abilities of the side chains to CB7. The conjugated oligomer TTQ (compound **6**) with a D-A-D structure was firstly synthesized by a Stille coupling reaction using compound **5** and tributyl(thieno[3,2-b]thiophen-2-yl)stannane as the starting materials, and their side chains were then quaternized with two different groups (*N*,*N*-dimethyladamantylamine group and trimethylamine group), producing TTQAd and TTQTMA, respectively. The chemical structures of all of the intermediates and the final products were confirmed by nuclear magnetic resonance spectroscopy (^1^H NMR and ^13^C NMR) and high-resolution mass spectrometer (HRMS) (Figures S1–S18).

### 2.2. Photophysical Properties of TTQAd and TTQTMA

The UV-Vis-NIR absorption spectra and fluorescence emission spectra of TTQAd and TTQTMA in DMSO and phosphate buffer solution (PBS) are shown in Figure 1a,b. Both TTQAd and TTQTMA had a maximum absorption peak at 672 nm in DMSO, benefiting from its D-A-D conjugated structure (Figure 1a). The absorption spectra of TTQAd and TTQTMA in PBS showed an apparent red-shift with a maximum absorption peak at 700 nm, with the tail extending to 900 nm (NIR I region). Both molecules had weak fluorescence in DMSO with an emission peak at 800 nm and fluorescence quenching in the aqueous solution due to the π-π stacking in a poor solvent (Figure 1b). Upon the addition of CB7, there is no significant shift in the peak of the absorption spectra and no significant change in the fluorescence emission spectra. This is due to the long distance between the terminal groups on the side chains and the main chain of the molecule. Therefore, the binding of CB7 to the terminal groups did not affect the optical property of the main chain (Figure 1a,b). In addition, TTQAd and TTQTMA have superior photostability compared to the commercial photosensitizer ICG. After 10 min of exposure to an 808 nm laser, the absorbance of TTQAd and TTQTMA decreased by less than 10%, whereas that of ICG decreased by 60% (Figure 1c).

### 2.3. ROS Generation Abilities of TTQAd and TTQTMA

As a photosensitizer, its ability to generate ROS directly determines its antimicrobial effect. The production of ROS by TTQAd and TTQTMA when exposed to NIR light was examined using various indicators (Figure 1d–f). Firstly, the total amount of ROS was assessed using 2′,7′-dichlorodihydrofluorescein (DCFH) as an indicator. DCFH can be transformed to a highly fluorescent DCF after oxidation by several ROS species, including singlet oxygen (^1^O_2_), hydroxyl radical, and superoxide anion. Figure 1d shows that both molecules were able to produce ROS when irradiated with an 808 nm laser, with a much higher ROS level than the commercial photosensitizer ICG. The addition of CB7 resulted in an increased capability of TTQAd to generate ROS, with no significant change in TTQTMA. 9,10-anthracenediyl-bis(methylene)dimalonic acid (ABDA) is an indicator for the determination of ^1^O_2_ (Figure 1e). ABDA can be oxidized by ^1^O_2_ and subsequently degraded, a reaction monitored by spectrophotometric recording of the decrease in optical density at 380 nm. The results demonstrated that both TTQAd and TTQTMA had a stronger ability to produce ^1^O_2_ than ICG. To further confirm that, singlet oxygen sensor green (SOSG) was utilized instead of ABDA for the detection of ^1^O_2_ (Figure 1f). SOSG emits blue fluorescence, but when selectively oxidized by ^1^O_2_, it forms endoperoxides emitting green fluorescence with an enhanced fluorescence intensity at 525 nm. The results were almost identical to those using ABDA, confirming the stronger ROS generation ability of TTQAd and TTQTMA than commercial photosensitizer ICG.

### 2.4. Self-Assembly Behavior of TTQAd and TTQTMA

**Figure 2:**
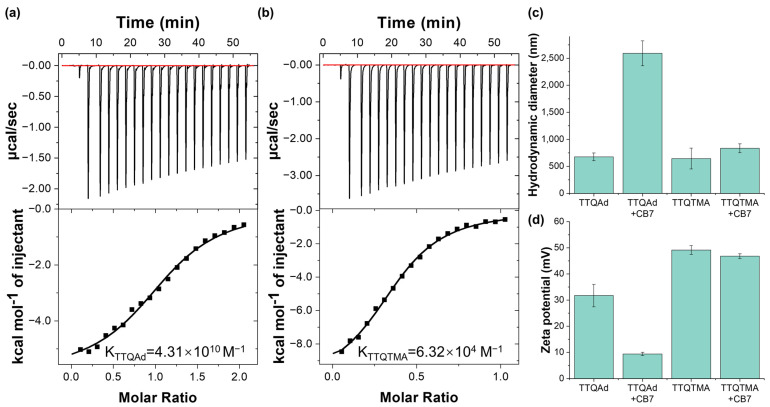
(**a**) The ITC isotherms and binding constants of CB7 titrated with TTQAd. (**b**) The ITC isotherms and binding constants of CB7 titrated with TTQTMA. (**c**) Diagram of the hydrodynamic diameters of TTQAd and TTQTMA before and after the addition of CB7 (TTQAd/TTQTMA:CB7 = 1:2). (**d**) Diagram of the zeta potentials of TTQAd and TTQTMA before and after the addition of CB7 (TTQAd/TTQTMA:CB7 = 1:2).

To assess the difference of the self-assembly behavior of TTQAd and TTQTMA with CB7, the isothermal titration calorimetry (ITC) experiments were firstly carried out. As shown in Figure 2a,b, the calculated binding constant for TTQAd with CB7 was *K*_a_ = 4.31 × 10^10^  M^−1^, while for TTQTMA with CB7, it was determined as *K*_a_ = 6.32 × 10^4^ M^−1^, indicating that the binding ability of TTQAd with CB7 was significantly stronger than that of TTQTMA, consistent with our expectations.

Secondly, the formation of the supramolecular complex was investigated by dynamic light scattering (DLS) measurements. Since both molecules have a hydrophobic backbone and hydrophilic side chains, they could spontaneously form nanoassemblies in aqueous solution. As indicated by Figure 2c, the hydrodynamic diameter of TTQAd and TTQTMA were determined to be 675.3 nm and 644.2 nm, respectively (Figure 2c and Figure S19). Both of them exhibited positive zeta potentials due to the presence of quaternary ammonium groups. In addition, the zeta potential of TTQAd (31.7 mV) was lower than that of TTQTMA (49.2 mV) (Figure 2d). This difference is likely attributed to the hydrophobic nature of the adamantane group which partially hindered the exposure of quaternary ammonium groups on the surface of the nanoassemblies. Upon the addition of CB7, TTQAd was able to form stable host-guest self-assemblies with CB7. The positive charge of the terminal groups of TTQAd was then largely shielded by CB7, leading to a further aggregation of the nanoassemblies. Therefore, the zeta potential of the supramolecular complex TTQAd-CB7 (9.4 mV) was much lower than that of TTQAd (31.7 mV), while the hydrodynamic diameter (2591.4 nm) was much larger than that of TTQAd (675.3 nm) (Figure 2c,d and Figure S19). In the case of TTQTMA, due to the much lower binding affinity of TTQTMA with CB7, the size and zeta potential of TTQTMA did not undergo significant changes following the addition of CB7. These results demonstrated that the ultrastable host-guest interaction between TTQAd and CB7 enabled the regulation of the aggregation behavior of TTQAd in aqueous solution.

### 2.5. Antibacterial Efficacy of TTQAd and TTQTMA

**Figure 3:**
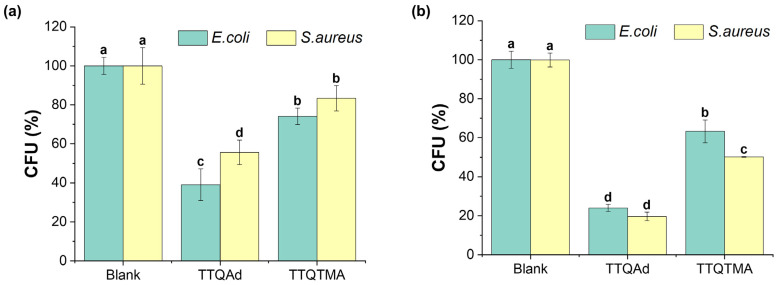
(**a**) Bacterial viabilities of *E. coli* and *S. aureus* treated with TTQAd/TTQTMA (20 μM) in the dark. (**b**) Bacterial viabilities of *E. coli* and *S. aureus* treated with TTQAd/TTQTMA (20 μM) followed by 808 nm laser irradiation (0.5 W cm^−2^, 10 min). Different letters (a, b, c, d) in the bar graphs represent significant difference at *p* < 0.05 levels.

Subsequently, the plate counting assay was used to evaluate the antibacterial capability of TTQAd and TTQTMA against the Gram-negative bacteria *Escherichia coli* (*E. coli*) and Gram-positive bacteria *Staphylococcus aureus* (*S. aureus*), and the results are shown in Figure 3 and Figure S20. The survival rates of *E. coli* and *S. aureus* were 39.1% and 55.7%, respectively, after incubation with 20 μM of TTQAd in the absence of light irradiation. After incubation with TTQTMA, the survival rates of *E. coli* and *S. aureus* were 74.1% and 83.4%, respectively (Figure 3a and Figure S20). These outcomes indicate that both TTQAd and TTQTMA are more toxic to *E. coli* than to *S. aureus* in the absence of light irradiation. This difference in toxicity may be attributed to the relatively thin and soft peptidoglycan layer present in the cells of Gram-negative *E. coli*, which does not effectively act as a barrier to the nanoassemblies of TTQAd and TTQTMA. On the other hand, the thicker and tougher peptidoglycan layer in Gram-positive bacteria, such as *S. aureus*, effectively shields them from entering into the bacterial interior [37].

Following exposure to an 808 nm laser (0.5 W cm^−2^, 10 min), the survival rates of *E. coli* and *S. aureus* treated with TTQAd were 24.0% and 19.6%, respectively, representing reductions of 25% and 65% compared to those in the dark. Meanwhile, the survival rates of *E. coli* and *S. aureus* treated with TTQTMA were 63.3% and 50.1%, respectively (Figure 3b and Figure S20), decreased by 16% and 40% compared to those in the dark. The effect of TTQAd either in the dark or with phototoxicity against both strains was stronger than that of TTQTMA due to the stronger lipophilic nature of the side chains of TTQAd compared to TTQTMA, resulting in easier passage across the cell membrane [38]. It is worth mentioning that the phototoxicity of TTQAd and TTQTMA arose from the photodynamic property, not a photothermal effect. To verify that, the photothermal properties of TTQAd and TTQTMA were tested. As shown in Figure S21, the molecules reached a maximum temperature of 35 °C under the irradiation of the 808 nm laser (0.5 W cm^−2^, 10 min), which was not sufficient to kill the bacteria, so all of the deaths of the TTQAd/TTQTMA-treated bacteria after laser irradiation were caused by the ROS generated.

To better present the aPDT effects of TTQAd and TTQTMA, a significance analysis was performed on the reduction in colony-forming units (CFU) before and after light exposure, as depicted in Figure S22. Under the treatment of TTQAd, a significant difference was observed in CFU reduction in both *E. coli* and *S. aureus* when comparing conditions of darkness to those of light exposure. In contrast, treatment with TTQTMA demonstrated a pronounced decrease in CFU count for *S. aureus* following light exposure, whereas *E. coli* did not exhibit a statistically significant change in CFU numbers before and after light exposure. This further indicates that TTQAd exhibited a stronger light toxicity against *E. coli* and *S. aureus* compared to TTQTMA.

### 2.6. Supramolecular Switch of the Antibacterial Efficacy of TTQAd

**Figure 4:**
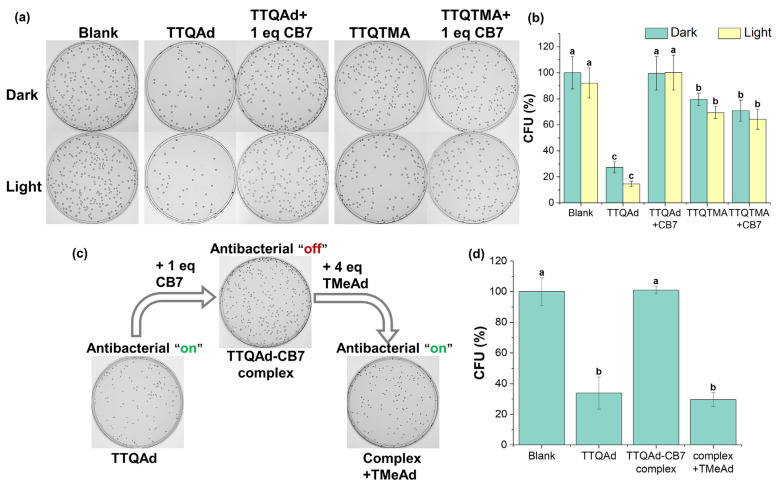
(**a**,**b**) Photographs of *E. coli* colonies on agar plates and bacterial viabilities of *E. coli* treated with TTQAd or TTQTMA (20 μM) before and after the addition of CB7 (40 μM) with/without 808 nm laser irradiation (0.5 W cm^−2^, 10 min). (**c**,**d**) Supramolecular switch of the antibacterial efficacy of TTQAd upon the treatment of CB7 and a subsequent treatment of competing guests TMeAd. TTQAd:CB7:TMeAd = 1:2:8. Different letters (a, b, c) in the bar graphs represent significant difference at *p* < 0.05 levels.

In order to verify the host-guest interactions on the regulation of TTQAd’s antibacterial ability, we first investigated the “turn-off” effect of CB7 on the antimicrobial activity of TTQAd (Figure S23 and Figure 4). We treated different amounts of CB7 to TTQAd (20 μM) and then tested the changes in the antibacterial activity of TTQAd against *E. coli*. The results showed that the complexation of TTQAd with only one equivalent of CB7 (40 μM) completely shielded the antibacterial activity of TTQAd and the bacterial viability was almost 100%. In contrast, one equivalent of CB7 could not shield the antibacterial activity of TTQTMA (Figure 4a,b). These, combined with the DLS results, revealed that the formation of large aggregates and the decrease in the surface zeta potentials of TTQAd upon the treatment of CB7 prevented the contact of TTQAd with bacteria.

Subsequently, the “turn-on” of the antibacterial activity of TTQAd after the disassembly of the supramolecular complex TTQAd-CB7 was investigated (Figure S24 and Figure 4c,d). Following the treatment of different amounts of competing guests (TMeAd) to TTQAd-CB7, the plate counting assay was performed. As a result, with the addition of four equivalents of TMeAd (160 μM), the toxicity of the TTQAd against *E. coli* was restored to that prior to the addition of CB7 (bacterial viability of 32.7%). It is worth mentioning that the competing guest TMeAd itself was not significantly toxic to *E. coli* (Figure S25), suggesting that the antibacterial activity exhibited after the treatment of TMeAd only originated from TTQAd displaced by the competing guests (Figure 4c,d).

### 2.7. Supramolecular Switch of the Antibacterial Efficacy of ICG

To highlight the advantages of TTQAd over a commercial photosensitizer in the regulation of antibacterial efficacy properties based on supramolecular strategies, we conducted antibacterial experiments to evaluate the killing effect of the commercially available photosensitizer ICG on *E. coli* and *S. aureus*, as illustrated in Figure S26. Under identical antibacterial conditions to those for TTQAd (20 μM), ICG manifested a negligible bactericidal effect on *E. coli*, irrespective of concomitant or absent CB7 treatment. However, ICG demonstrated significant toxicity towards *S. aureus*, achieving an approximately 98% bacterial reduction at a 1 μM concentration when illuminated. Upon the addition of one equivalent of CB7, the antibacterial efficiency of ICG diminished, with the bactericidal rate dropping from 98% to 78%. However, the antibacterial action of ICG could not be entirely shielded by CB7; ICG retained effectiveness in comparison to control samples in the absence of light exposure. Overall, in comparison to TTQAd, ICG demonstrates a worse photostability, a negligible bactericidal effect on *E. coli*, and is effective on *S. aureus* but not reversibly regulated by CB7.

### 2.8. Biocompatibility Evaluations

**Figure 5:**
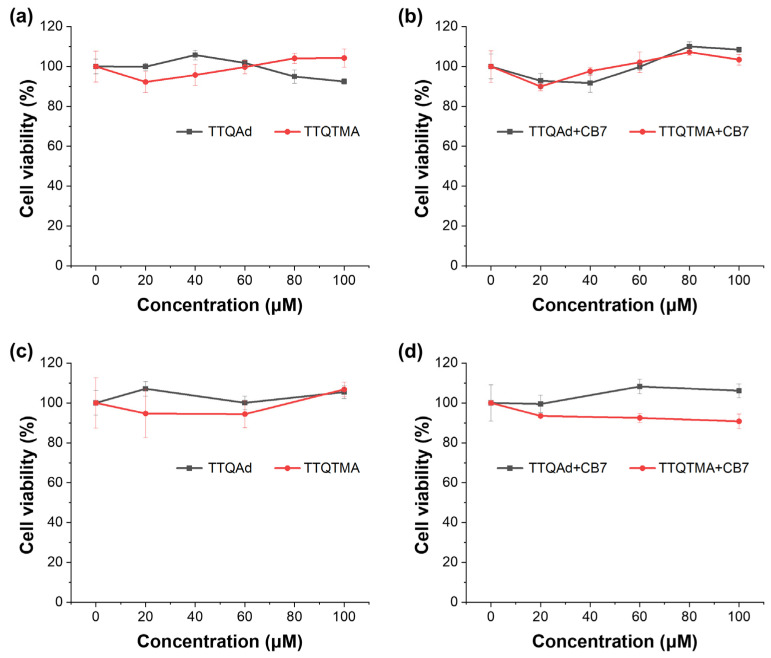
Cell viability after treating with TTQAd (**a**), TTQTMA (**a**), TTQAd + CB7 (**b**), and TTQTMA + CB7 (**b**) at different concentrations in the absence of light. Cell viability after treating with TTQAd (**c**), TTQTMA (**c**), TTQAd + CB7 (**d**), and TTQTMA + CB7 (**d**) at different concentrations followed by 808 nm laser irradiation (0.5 W cm^−2^).

In order to assess the biocompatibility of all of the compounds utilized in the supramolecular regulation systems, standard methylthiazolyltetrazolium (MTT) assays were performed against human embryonic kidney cells HEK 293. HEK 293 cells were incubated with different concentrations of TTQAd, TTQTMA, and their supramolecular complexes with CB7, respectively, for 24 h. As shown in the results, the molecules exhibited negligible toxicity to the cells before and after the addition of CB7 both in the dark and under 808 nm laser irradiation (0.5 W cm^−2^, 10 min), suggesting their good biocompatibility (Figure 5). The cytotoxicity assessments of CB7 and the competitor TMeAd were also conducted, respectively. CB7 exhibited excellent biocompatibility, whereas TMeAd showed increased cytotoxicity at higher concentrations due to its positive charge and small molecular weight (Figure S27). In spite of that, cell survival rates were still above 80% at the concentration of TMeAd (160 μM) used in the above antibacterial assay. Considering the much shorter incubation time (30 min) in the antibacterial assay, the cytotoxicity of TMeAd was negligible.

## 3. Materials and Methods

### 3.1. Chemicals and Instruments

Unless stated otherwise, all analytical grade chemicals and solvents in this paper were purchased from commercial vendors and used without further purification. Chemicals used in this study were purchased from Aladdin (Shanghai, China) or Sinopharm Chemical Reagent Co., Ltd. (Shanghai, China). ^1^H NMR spectra and ^13^C NMR spectra were recorded on a 400 MHz Bruker AVANCE NEO spectrometer (Bruker, Karlsruhe, Germany) and Bruker 600 MHZ AVANC NEO 600 spectrometer (Bruker, Karlsruhe, Germany) with tetramethylsilane (TMS; δ = 0 ppm) as the internal standard. High-resolution mass spectra (HRMS) were obtained on an AB Sciex TripleTOF 4600 mass spectrometer (AB Sciex, Framingham, MA, USA) and Bruker Solarix mass spectrometer (Bruker, Karlsruhe, Germany). UV-vis spectra were recorded on Agilent Cary 5000 (Agilent, Santa Clara, CA, USA) and fluorescence spectra (FL) were obtained from Perkinelmer LS55 (Perkinelmer, Waltham, MA, USA). The size distribution and zeta potential were tested on an Anton Paar GmbH Litesizer 500 (Anton Paar, Graz, Austria). The 808 nm laser (LR-MFJ-808/5000mW) and laser power meter (VLP-2000-10W) were purchased from Changchun Laser Techonology Co., Ltd. (Changchun, China). Photothermal conversion behaviors were monitored by the FLIR E8 IR camera (FLIR, Wilsonville, OR, USA). The instrument utilized in all ITC measurements was a MicroCal iTC200 (MicroCal, Cambridge, UK). *E. coli* ATCC 11229 and *S. aureus* ATCC 29213 were obtained from the China General Microbiological Culture Collection Center (CGMCC, Beijing, China). HEK 293 cells (CatLog No. CL-0001) were provided by Procell Life Science&Technology Co., Ltd. (Wuhan, China).

### 3.2. Synthesis of TTQAd and TTQTMA

4,7-Dibromo-2,1,3-benzothiadiazole-5,6-diamine (**1**). Compound **1** was prepared following a modified procedure from the previous literature [39]. To a stirred solution of 4,7-dibromo-5,6-dinitrobenzo[c][1,2,5]thiadiazole (1.9 g, 5 mmol) in glacial acetic acid (23 mL), fine iron powder (3.88 g, 60 mmol) was added portion wise at 0 °C. The reaction mixture was then stirred at ambient temperature for 12 h before being poured into ice-cold water, resulting in the formation of a precipitate that was subsequently filtered off. The obtained product was washed with water, yielding a yellow powder with an 87% yield.

^1^H NMR (400 MHz, DMSO-d6) δ 6.35 (s, 4H), 1.91 (s, 3H).

Dimethyl-1-adamantylamine (**2**). Compound **2** was prepared following a modified procedure of the previous literature [40]. 1-Adamantylamine (2.1 g, 14 mmol) was dissolved in formaldehyde (5 mL) and heated to 100 °C under reflux. Formic acid (1.5 mL) was added to the mixture dropwise using an addition funnel in the span of 1.5 h and the mixture was refluxed for another 3 h. Afterward, the solution was allowed to cool to room temperature and then adjusted to a pH of 12 using a sodium hydroxide solution. The product was extracted from the mixture with dichloromethane and dried with potassium carbonate. Following this, the dichloromethane solvent was removed using rotary evaporation, resulting in a colorless oily liquid with a yield of 79%.

^1^H NMR (400 MHz, Chloroform-d) δ 2.22 (s, 6H), 2.06–2.00 (m, 3H), 1.66–1.48 (m, 12H).

4,4′-Dihydroxy-benzil (**3**). Compound **3** was prepared following a modified procedure of the previous literature [41]. 4,4′-Dimethoxy-benzil (3.0 g, 10.9 mmol) and pyridinium hydrochloride (7.8 g, 67.2 mmol) were heated at 220 °C until the solid mixture completely melted. The heating was maintained for 1.5 h. After cooling to 80 °C, water was added dropwise to give a suspension, which was then filtered while hot. The collected solid was then dissolved in ethyl acetate and the solution was dried over magnesium sulfate. The ethyl acetate solvent was removed by rotary evaporation, giving a yellow powder with a yield of 90.6%.

^1^H NMR (400 MHz, DMSO-d6) δ 10.82 (s, 2H), 7.74 (d, *J* = 8.7 Hz, 4H), 6.92 (d, *J* = 8.7 Hz, 4H).

1,2-bis(4-((6-bromohexyl)oxy)phenyl)ethane-1,2-dione (**4**). 4,4′-Dihydroxy-benzil (1 g, 4.2 mmol) and K_2_CO_3_ (1.8 g, 12.7 mmol) in acetone (10 mL) were stirred at ambient temperature for 0.5 h under a nitrogen atmosphere. Then 1,6-dibromohexane (5.27 g, 21 mmol) was added into the flask. Afterward, the mixture was heated to 90 °C and maintained at that temperature for 12 h. After cooling to room temperature, the inorganic salt was filtered out, and the filtrate was concentrated using a rotary evaporator. The crude product was subsequently purified via silica gel column chromatography using a petroleum ether/ethyl acetate mixture (92:8 by volume) as an eluent. A white powder was ultimately obtained with a yield of 53%.

^1^H NMR (400 MHz, Chloroform-d) δ 7.93 (d, *J* = 8.9 Hz, 4H), 6.94 (d, *J* = 8.9 Hz, 4H), 4.04 (t, *J* = 6.4 Hz, 4H), 3.42 (t, *J* = 6.7 Hz, 4H), 1.86 (dp, *J* = 27.5, 6.7 Hz, 8H), 1.54–1.45 (m, 8H). ^13^C NMR (101 MHz, Chloroform-d) δ 193.55, 164.40, 132.41, 126.20, 114.71, 68.19, 33.72, 32.60, 28.85, 27.85, 25.20. HR-MS (LC-Q-TOF): *m*/*z* [M]^+^ cacld for C_26_H_32_Br_2_O_4_^+^, 568.0647; found, 568.0757.

4,9-dibromo-6,7-bis(4-((6-bromohexyl)oxy)phenyl)-[1,2,5]thiadiazolo[3,4-g]quinoxaline (**5**). Compound **1** (0.5898 g, 1.8 mmol) and compound **4** (1 g, 1.8 mmol) were dissolved in a mixture of toluene (15 mL) and AcOH (15 mL), and the resulting solution was heated to 112 °C and maintained at that temperature for 12 h. After cooling to room temperature, the mixture was poured into water and extracted with ethyl acetate. The organic extracts were washed with water until neutral pH and dried over anhydrous Na_2_SO_4_. The solvent was removed by vacuum distillation, and the resulting product was isolated via preparative thin-layer chromatography on silica gel using dichloromethane/petroleum ether (1/5) as an eluent, yielding a red solid with a 40.3% yield.

^1^H NMR (400 MHz, Chloroform-d) δ 7.76 (d, *J* = 8.8 Hz, 4H), 6.91 (d, *J* = 8.8 Hz, 4H), 4.03 (t, *J* = 6.4 Hz, 4H), 3.44 (t, *J* = 6.8 Hz, 4H), 1.92 (t, *J* = 6.9 Hz, 4H), 1.88–1.80 (m, 4H), 1.53 (d, *J* = 4.4 Hz, 8H). ^13^C NMR (151 MHz, Chloroform-d) δ 161.20, 155.47, 152.24, 138.12, 132.00, 130.11, 114.43, 113.35, 67.92, 33.78, 32.65, 29.00, 27.90, 25.28. HR-MS (LC-Q-TOF): *m*/*z* [M]^+^ cacld for C_32_H_32_Br_4_N_4_O_2_S^+^, 855.8939; found, 855.9046.

6,7-bis(4-((6-bromohexyl)oxy)phenyl)-4,9-bis(thieno[3,2-b]thiophen-2-yl)-[1,2,5]thiadiazolo[3,4-g]quinoxaline (**6**). Compound **5** (100 mg, 0.1 mmol), tributyl(thieno[3,2-b]thiophen-2-yl)stannane (100 mg, 0.22 mmol), and tetrakis(triphenylphosphine)palladium(0) (30 mg, 0.026 mmol) were transferred to a Schlenk flask under a nitrogen atmosphere. Then, toluene (5 mL) was added and the mixture was heated to 110 °C and maintained for 24 h. After cooling to room temperature, the solvent was removed using a rotary evaporator. The crude was then purified via silica gel column chromatography using a mixture of petroleum ether/ethyl acetate (85:15 by volume) as an eluent. The pure product was obtained as a dark green solid with a yield of 80%.

^1^H NMR (600 MHz, Chloroform-d) δ 9.29 (s, 2H), 7.71 (d, *J* = 8.6 Hz, 4H), 7.47 (d, *J* = 5.0 Hz, 2H), 7.33 (d, *J* = 5.1 Hz, 2H), 6.92 (d, *J* = 8.4 Hz, 4H), 4.05 (t, *J* = 6.4 Hz, 4H), 3.46 (t, *J* = 6.7 Hz, 4H), 1.94 (dd, *J* = 9.1, 4.7 Hz, 4H), 1.89–1.84 (m, 4H), 1.57 (d, *J* = 4.8 Hz, 8H). ^13^C NMR (151 MHz, Chloroform-d) δ 160.38, 152.75, 151.56, 145.33, 139.73, 138.68, 134.82, 132.44, 130.43, 128.92, 125.37, 121.01, 119.77, 114.16, 67.85, 33.84, 32.69, 29.13, 27.97, 25.36. HR-MS (LC-Q-TOF): *m*/*z* [M]^+^ cacld for C_44_H_38_Br_2_N_4_O_2_S_5_^+^, 973.9945; found, 974.0139.

TTQTMA. Compound **6** (10 mg, 0.01 mmol) was dissolved in a mixture of THF (0.5 mL) and acetonitrile (1 mL). Then trimethylamine solution in THF (2 M, 0.5 mL) was added under a nitrogen atmosphere. After the mixture was heated at 80 °C for 12 h, the solvent was removed using a rotary evaporator, and the product was obtained by precipitation in ethyl acetate. The product TTQTMA was afforded as a dark solid with a yield of almost 100%.

^1^H NMR (600 MHz, DMSO-d6) δ 9.18 (s, 2H), 7.82 (d, *J* = 5.1 Hz, 2H), 7.62–7.57 (m, 4H), 7.50 (d, *J* = 5.1 Hz, 2H), 6.97 (d, *J* = 8.3 Hz, 4H), 4.06 (t, *J* = 6.4 Hz, 4H), 3.35–3.29 (m, 6H), 3.07 (s, 18H), 1.81 (p, *J* = 6.6 Hz, 4H), 1.74 (dq, *J* = 12.0, 8.0, 6.1 Hz, 4H), 1.53 (p, *J* = 7.6 Hz, 4H), 1.39 (p, *J* = 7.6 Hz, 4H). ^13^C NMR (151 MHz, DMSO-d6) δ 153.00, 150.99, 145.46, 139.56, 138.23, 134.29, 132.62, 131.10, 130.33, 125.62, 120.56, 120.37, 114.54, 68.00, 65.77, 52.68, 51.37, 28.94, 26.01, 25.61, 22.52. HR-MS (LC-Q-TOF): *m*/*z* [M−2Br^−^]^2+^ cacld for C_50_H_56_N_6_O_2_S_5_^2+^, 466.1529; found, 466.1527.

TTQAd. Compound **6** (10 mg, 0.01 mmol) and compound **2** (36.78 mg, 0.2 mmol) were dissolved in anhydrous DMF (2 mL), and the mixture was heated at 80 °C for 12 h under a nitrogen atmosphere. Then, the solvent was removed using a rotary evaporator and the product was obtained by precipitation in ethyl acetate. The product TTQAd was afforded as a dark solid with a yield of almost 100%.

^1^H NMR (400 MHz, DMSO-d6) δ 9.23 (d, *J* = 1.5 Hz, 2H), 7.83 (d, *J* = 5.1 Hz, 2H), 7.65 (d, *J* = 8.4 Hz, 4H), 7.52 (d, *J* = 5.1 Hz, 2H), 7.01 (d, *J* = 8.4 Hz, 4H), 4.08 (t, *J* = 6.5 Hz, 4H), 3.23–3.14 (m, 4H), 2.84 (s, 12H), 2.23 (s, 6H), 2.04 (d, *J* = 3.0 Hz, 12H), 1.81 (dd, *J* = 14.8, 7.6 Hz, 8H), 1.66–1.60 (m, 12H), 1.48 (dt, *J* = 55.4, 7.8 Hz, 9H). ^13^C NMR (151 MHz, DMSO-d6) δ 160.40, 152.77, 151.57, 145.34, 139.75, 138.70, 134.83, 132.45, 130.44, 128.93, 125.39, 121.02, 119.78, 114.18, 67.85, 33.84, 32.69, 29.13, 27.97, 25.36. HR-MS (LC-Q-TOF): *m*/*z* [M−2I^−^]^2+^ cacld for C_68_H_80_N_6_O_2_S_5_^2+^, 586.2468; found, 586.2453.

### 3.3. Evaluation of ROS Generation Efficiency

#### 3.3.1. Determination of Total Amount of ROS Using DCFH

A 2′,7′-dichlorodihydrofluorescein diacetate (DCFH-DA, 1 mM) stock solution was obtained by dissolving 2.7 mg DCFH-DA in 10 mL of ethanol. A total of 0.5 mL of 1 mM DCFH-DA stock solution was mixed with 2 mL of 0.01 M NaOH aqueous solution, and then sat at room temperature for 30 min in the dark to obtain DCFH. Afterward, the prepared DCFH solution was diluted with 1 × PBS (10 mM, pH = 7.4) to a final concentration of 10 μM. Then the DCFH solution was mixed with an equal volume of PBS, ICG, TTQAd, TTQAd + CB7, TTQTMA, and TTQTMA + CB7, respectively, to obtain a final concentration of 5 μM of the test molecule, 100 μM of CB7, and 5 μM of DCFH. A total of 200 μL of the above mixture was transferred into a black 96-well plate, and the prepared solution was irradiated using an 808 nm laser with a light intensity of 0.1 W cm^−2^ for 10 min, and the fluorescence intensity at 525 nm (Ex = 488 nm) was recorded using a microplate reader every 2 min.

#### 3.3.2. Determination of Singlet Oxygen (^1^O_2_) Production Using ABDA

A total of 1 mg of ABDA powder was dissolved in 100 μL of DMSO to obtain a stocking solution of 25 mM, which was then diluted to 100 μM using water. Equal volumes of water, ICG, TTQAd, TTQAd + CB7, TTQTMA, and TTQTMA + CB7 were then mixed with ABDA solution, respectively. The final concentrations of the test molecules were 5 μM for TTQAd or TTQTMA, 100 μM for CB7, and 50 μM for ABDA. A total of 200 μL of the above mixture solution was transferred into a transparent 96-well plate, and the solution was irradiated using an 808 nm laser with a light intensity of 0.1 W cm^−2^ for 10 min. The absorption spectra in the range of 300–600 nm were recorded using a microplate reader every 2 min.

#### 3.3.3. Determination of ^1^O_2_ Production Using SOSG

An SOSG (1 mM) stock solution was obtained by dissolving 100 μg SOSG in 33 μL of methanol and then diluted to 10 μM with 1 × PBS. Equal volumes of PBS, ICG, TTQAd, TTQAd + CB7, TTQTMA, and TTQTMA + CB7 were then mixed with SOSG solution, respectively, to obtain final concentrations of 5 μM of the test molecule, 100 μM of CB7, and 5 μM of SOSG. A total of 200 μL of the above mixture solution was transferred into a black 96-well plate, and then irradiated using an 808 nm laser with a light intensity of 0.1 W cm^−2^ for 10 min, and the fluorescence intensity at 525 nm (Ex = 488 nm) was recorded using a microplate reader every 2 min.

### 3.4. The Binding Constants of TTQAd and TTQTMA with CB7

ITC experiments were performed at 25 °C. The binding constant of TTQTMA to CB7 was determined by direct titration method. The binding constant of TTQAd to CB7 was too high to be measured accurately by direct ITC titration and therefore was measured by the multistep competition method.

The binding constants of TTQTMA to CB7 were measured as follows. An aqueous solution (0.2 mM) of CB7 was placed in the sample cell, to which a solution of TTQTMA (1 mM) was added stepwise in a series of 20 injections (2 μL each). The heat of dilution was measured by injecting the guest solution into a blank solution without CB7, and the net heat effect was obtained by subtracting this value from the overall heat effect observed. The titration results were curve fitted using a single set of identical site models.

The binding constants of TTQAd to CB7 were measured by competitive titration. The competitive titration method was performed employing three appropriate competitive guests for titration. In this experiment, L-phenylalanine hydrochloride, 1,6-hexanediamine hydrochloride, and aminomethylcyclohexane hydrochloride were chosen as competitive guests to determine the binding constants of TTQAd. The procedure for testing the binding constant of L-phenylalanine hydrochloride to CB7 is consistent with that described above for TTQTMA. For other molecules, the binding constants to CB7 were determined by competitive titration methods. For example, the binding constant of 1,6-hexanediamine hydrochloride was measured using L-phenylalanine hydrochloride as competitor guest. In particular, 1,6-hexanediamine hydrochloride (1 mM) was titrated into CB7 (0.1 mM) in water in the presence of L-phenylalanine (0.2 mM), and the heat of dilution was determined by titration of the 1,6-hexanediamine hydrochloride (1 mM) into water. Likewise, the binding constant of aminomethylcyclohexane hydrochloride was measured using 1,6-hexanediamine hydrochloride as competitor guest. Finally, the binding constant of TTQAd was determined by using aminomethylcyclohexane hydrochloride as competitor guest. TTQAd (1 mM) was used to titrate CB7 (0.1 mM) in water in the presence of aminomethylcyclohexane hydrochloride (0.2 mM). The heat of dilution was measured by injecting the guest solution into a blank solution without CB7. All competitive titration results were curve fitted using a competitive binding model.

### 3.5. The Hydrodynamic Diameter and Zeta Potential of TTQAd and TTQTMA before and after the Addition of CB7

The hydrodynamic particle size and zeta potential of the nanoparticles were determined by dynamic light scattering, and the test molecule was diluted with distilled water to obtain a final concentration of 10 μM (with or without 20 μM of CB7), and measured three times at 25 °C.

### 3.6. Bacterial Culture

A single *E. coli* colony or *S. aureus* colony was aseptically transferred to 10 mL of fresh LB medium and then incubated at 37 °C for 8 h. When the bacterial solution was turbid, it was centrifuged at 8000 rpm for 2 min at room temperature, the supernatant was discarded, it was washed twice with PBS, and then the concentration of the bacteria was adjusted to 2.0 × 10^7^ CFU mL^−1^ for the subsequent experiments.

### 3.7. Evaluation of the Antibacterial Effect of TTQAd, TTQTMA against E. coli and S. aureus

The bacterial solution was mixed with 40 μM of TTQAd and TTQTMA in equal volumes. The final concentration of TTQAd or TTQTMA was 20 μM, and the bacterial concentration was 1.0 × 10^7^ CFU mL^−1^. The bacterial solution was then incubated at 37 °C in the dark for 30 min, followed by irradiation using an 808 nm laser (0.5 W cm^−2^) for 10 min. Afterward, the bacterial solution was diluted 2 × 10^3^ times with PBS, and then 100 μL was taken and spread on an agar plate. The CFU were recorded after 18~20 h of incubation.

### 3.8. Evaluation of the Supramolecular “Switch-off” Effects of CB7 on the Antibacterial Ability of TTQAd

TTQAd solution was prepared at a concentration of 80 μM, and the same volume of CB7 at concentrations of 0, 80, 160, 320, 640, and 800 μM was added, respectively. To the above mixture, an equal volume of *E. coli* solution was added to make a final concentration of 20 μM of TTQAd and 1.0 × 10^7^ CFU mL^−1^ of *E. coli*, while the final concentrations of CB7 were 0, 20, 40, 80, 160, and 200 μM, respectively. Then the bacterial solution was incubated at 37 °C for 30 min, followed by irradiation using an 808 nm laser (0.5 W cm^−2^) for 10 min. The bacterial solution was subsequently diluted 2 × 10^3^ times with PBS, and 100 μL was spread on an agar plate. The CFU were recorded after 18~20 h of incubation.

### 3.9. Comparison of the “Switch-off” Effects of CB7 on TTQAd and TTQTMA

The solutions of TTQAd-CB7 and TTQTMA-CB7 were firstly prepared and then mixed with an equal volume of *E. coli* solution to make a final concentration of 20 μM of TTQAd and TTQTM, 40 μM of CB7, and 1.0 × 10^7^ CFU mL^−1^ of *E. coli*. After incubation at 37 °C for 30 min, the dark group was placed in the dark, and the light group was irradiated using an 808 nm laser for 10 min (0.5 W cm^−2^). Then the bacterial solution was diluted 2 × 10^3^ times with PBS, and 100 μL was taken and spread on a 90 mm agar plate. The CFU were recorded after incubation for 18–20 h.

### 3.10. Evaluation of the Toxicity of TMeAd to E. coli

TMeAd molecules at concentrations of 0, 80, 160, 240, 320, and 400 μM were prepared, and mixed with an equal volume of *E. coli* solution. The final concentrations of TMeAd molecules were 0, 40, 80, 120, 160, and 200 μM, and the bacterial concentration was 1.0 × 10^7^ CFU mL^−1^. The incubation was carried out at 37 °C for 30 min and then diluted 2 × 10^3^ times with PBS, and 100 μL of the solution was spread on a 90 mm agar plate, and the CFU were recorded after incubation for 18–20 h.

### 3.11. Supramolecular “Switch-on” Effects of TMeAd on the Antibacterial Ability of TTQAd-CB7 Supramolecular Complexes

TTQAd-CB7 supramolecular complex solution (160 μM) was prepared by mixing an equal volume of TTQAd solution (160 μM) with CB7 solution (320 μM). Afterward, TMeAd solutions with concentrations of 0, 160, 320, 480, 640, and 800 μM were prepared and mixed with TTQAd-CB7 solution in equal volumes, and then mixed with *E. coli* solution. After incubation at 37 °C for 30 min, the bacterial solution was diluted 2 × 10^3^ times with PBS, 100 μL of the solution was spread on a 90 mm agar plate, and the CFU were recorded after incubation for 18–20 h.

### 3.12. Evaluation of the Antibacterial Effect of TTQAd, TTQTMA against E. coli and S. aureus

Due to the distinct bactericidal effects of ICG on *E. coli* and *S. aureus*, varying concentrations were employed for the experiments. ICG solution was prepared at a concentration of 80 μM, and the same volume of CB7 at a concentration of 160 μM was added. To the above mixture, an equal volume of *E. coli* solution was added to make a final concentration of 20 μM of ICG and 1.0 × 10^7^ CFU mL^−1^ of *E. coli*, while the final concentration of CB7 was 40 μM, respectively. Then the bacterial solution was incubated at 37 °C for 30 min, followed by irradiation using an 808 nm laser (0.5 W cm^−2^) for 10 min. For *S. aureus*, the final test concentrations are 1 μM for ICG and 2 μM for CB7, and the bacterial solution concentration is 1.0 × 10^8^ CFU mL^−1^. The remaining conditions are consistent with those applied to *E. coli.*

### 3.13. Cytotoxicity Test

Human embryonic kidney cells, HEK 293, were selected to determine the cytotoxicity of the molecules used. HEK 293 cells were cultured in DMEM complete medium supplemented with 10% fetal bovine serum. Prior to the experiment, cells in optimal condition were trypsinized, subjected to a 10-fold dilution for cell counting, and then seeded into 96-well plates at a density of 8 × 10^3^ cells per well. The cells were then incubated for 24 h at 37 °C with 5% CO_2_. Then, TTQAd, TTQAd + CB7, TTQTMA, and TTQTMA + CB7 solutions in culture medium were added to the cells with final concentrations of 0, 20, 40, 60, 80, and 100 μM, respectively. After 30 mins incubation at 37 °C, the cells were either kept in the dark or irradiated with an 808 nm laser (0.5 W cm^−2^) for 10 min per well. After incubating at 37 °C for 24 h, the cells were treated with 10 μL of MTT solution (5 mg mL^−1^ in PBS) per well and incubated for an additional 4 h. The MTT solution was then discarded and 100 μL of DMSO was added to each well, followed by vigorous shaking for 5 min to dissolve the purple formazan crystals. The absorbance of the DMSO solution at 570 nm was measured using a microplate reader.

### 3.14. Statistical Analysis

The data were presented as the mean ± standard deviations (SD) via triplicate experiments. Statistical analysis was performed using a one-way analysis of variance (ANOVA). Differences were considered statistically significant at the 95% confidence level (*p* < 0.05).

## 4. Conclusions

In summary, we have designed and synthesized two conjugated oligoelectrolytes, TTQAd and TTQTMA, which possessed absorption wavelengths that are longer than those of most photosensitizers and the ability to generate ROS when exposed to 808 nm laser irradiation. Due to the different cationic side chains, these two photosensitizers demonstrated varying effects on bactericidal activity. In particular, TTQAd exhibited a stronger dark and light toxicity against *E. coli* and *S. aureus* than TTQTMA. Moreover, TTQAd showed a stronger toxicity against *E. coli* in the absence of light, and against *S. aureus* in the presence of light, making it possible to selectively combat both bacterial strains under different treatment conditions. Furthermore, benefiting from the cationic adamantaneammonium group on the side chains, TTQAd was more likely to bind to CB7 to shield its cationic terminal groups and prevent direct contact with the bacteria, turning off the antibacterial effect. Consequently, with the introduction of competing guests, TTQAd could be released, restoring its antibacterial effect, and the antibacterial effect is turned on. This design significantly lowered the required concentration of CB7 (1 eq) and the competitive guest (4 eq). Therefore, the selective antibacterial effect against different bacterial strains can be achieved by a rational design of the side chains of the conjugated oligoelectrolytes, and the introduction of guest moieties on the side chains can further allow for the reversible regulation of the antibacterial effect using a host-guest self-assembly strategy. The latter strategy can also effectively prevent a prolonged exposure of the photosensitizers to bacteria, allowing for the controlled release of active photosensitizers, and showing promise in preventing the emergence of drug-resistant bacteria.

Despite the above findings, there are still some limitations in this work. One particular concern is the suboptimal antibacterial efficacy of TTQAd, which is less than 1 log in bacterial reduction. Efforts are underway to refine the molecular structure in pursuit of augmented antibacterial efficiency; for example, we are modifying the donor-acceptor configurations within the conjugated backbone to enhance the photodynamic antibacterial properties.

## Data Availability

The data presented in this study are available in article and Supplementary Materials.

## Supplementary Materials

The following supporting information can be downloaded at: https://www.mdpi.com/article/10.3390/molecules29051040/s1. Scheme S1: The synthetic routes of TTQAd and TTQTMA; Figures S1–S18: The ^1^H NMR, ^13^C NMR, and HRMS results of all compounds; Figure S19: The hydrodynamic diameter histogram of TTQAd and TTQTMA before and after the addition of CB7; Figure S20: The photographs of plate counting assay for antibacterial efficacy of TTQAd and TTQTMA; Figure S21: Photothermal conversion of TTQAd and TTQTMA in PBS (20 μM) under 808 nm laser irradiation (0.5 W cm^−2^, 10 min); Figure S22: Bacterial viabilities of *E. coli* (a) and *S. aureus* (b) treated with TTQAd/TTQTMA (20 μM) with/without 808 nm laser irradiation (0.5 W cm^−2^, 10 min). Figure S23: Photographs of *E. coli* colonies on agar plates and bacterial viabilities of *E. coli* treated with TTQAd before and after the addition of different amounts of CB7 with/without 808 nm laser irradiation (0.5 W cm^−2^); Figure S24: Photographs of *E. coli* colonies on agar plates and bacterial viabilities of *E. coli* treated with TTQAd-CB7 and different amounts of TMeAd; Figure S25: Toxicity of TMeAd against *E. coli*; Figure S26: The photographs of plate counting assay and bacterial viabilities for antibacterial efficacy of ICG; Figure S27: Biocompatibility of CB7 and TMeAd.
