# Supplementary material for: Supramolecular Switch for the Regulation of Antibacterial Efficacy of Near-Infrared Photosensitizer
Source: Molecules. 2024 Feb 28;29(5):1040. doi: 10.3390/molecules29051040 (PMC10935030; doi:10.3390/molecules29051040)
Supplement: Supplementary file 1 [file molecules-29-01040-s001.zip › molecules-2852966-supplementary.pdf]

Supporting Information

Supramolecular Switch for the Regulation of Antibacterial Efficacy of Near-Infrared Photosensitizer

Yu-Na Jiang [1,2], Manqi Tan [1,2], Chenglong He [1,2], Jiaxi Wang [3], Yi Wei [3], Ningning Jing [4], Bing Wang [2,5], Fang Yang [1,2,5,*], Yujie Zhang [1,2,5,*] and Meng Li [2,5,*]

[1] Cixi Biomedical Research Institute, Wenzhou Medical University, Ningbo 315302, China; jiangyuna@nimte.ac.cn (Y.-N.J.); tanmanqi@nimte.ac.cn (M.T.); hechenglong@nimte.ac.cn (C.H.)
[2] Ningbo Key Laboratory of Biomedical Imaging Probe Materials and Technology, Ningbo Institute of Materials Technology and Engineering, Chinese Academy of Sciences, Ningbo 315201, China; wangbing@nimte.ac.cn
[3] School of Materials Science and Engineering, University of Science and Technology Beijing, Beijing 100083, China; jiaxi0726@163.com (J.W.); weiyi5166@126.com (Y.W.)
[4] College of Science and Technology, Ningbo University, Ningbo 315300, China; jingnn7009@163.com
[5] Zhejiang International Scientific and Technological Cooperative Base of Biomedical Materials and Technology, Ningbo Cixi Institute of Biomedical Engineering, Ningbo 315300, China
* Correspondence: yangf@nimte.ac.cn (F.Y.); zhangyujie@nimte.ac.cn (Y.Z.); limeng@nimte.ac.cn (M.L.)

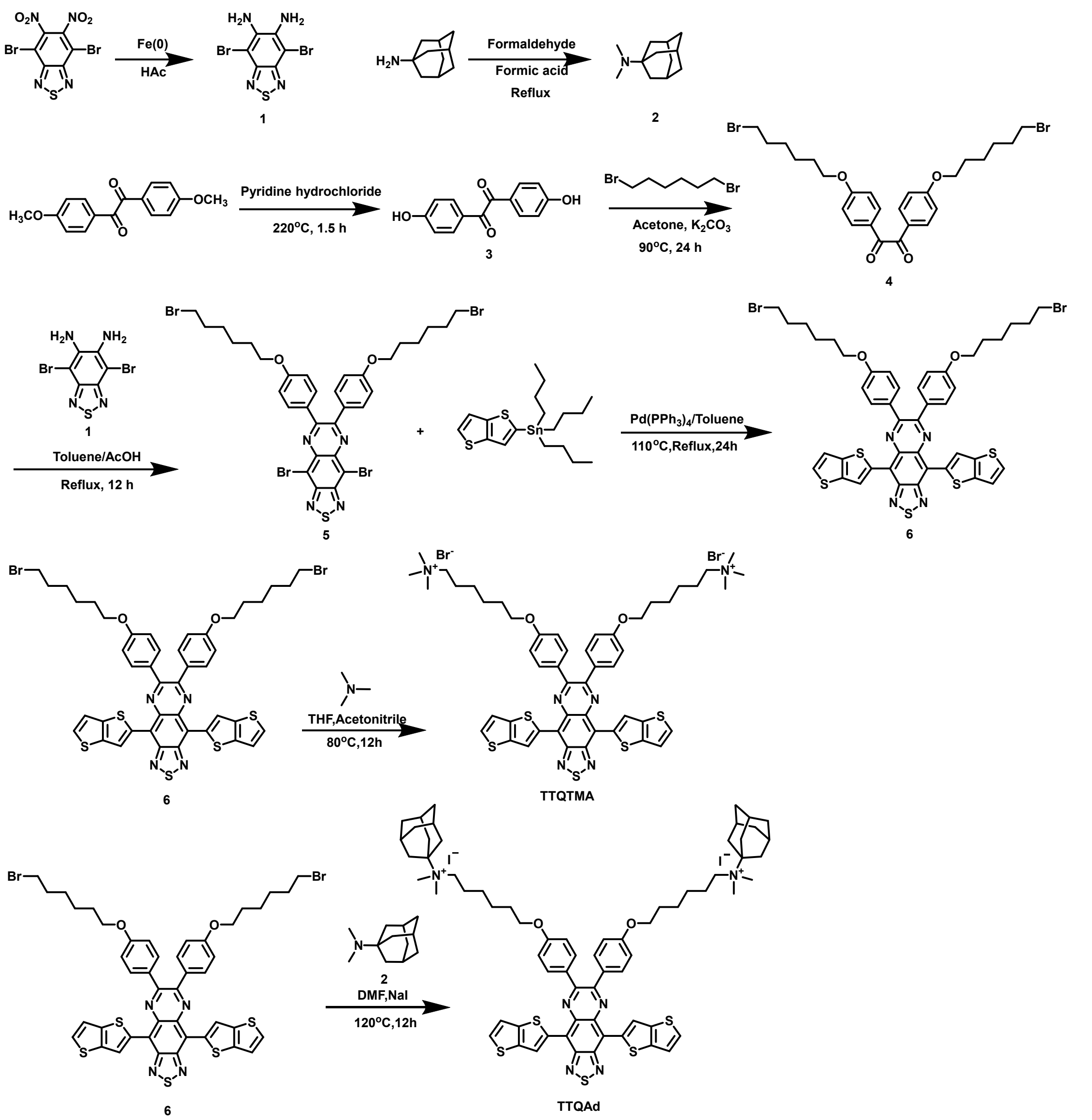

Scheme S1. The synthetic routes of TTQAd and TTQTMA.

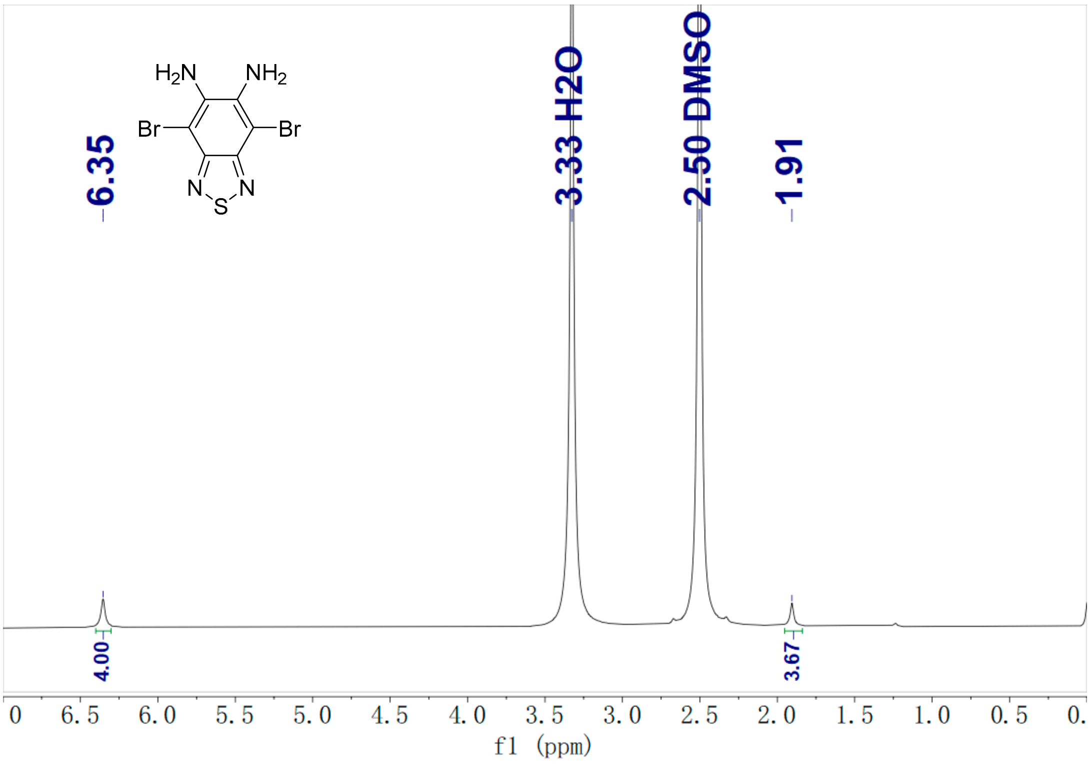

Figure S1. ^{1}H NMR spectrum of 4,7-dibromo-2,1,3-benzothiadiazole-5,6-diamine (1) in DMSO.

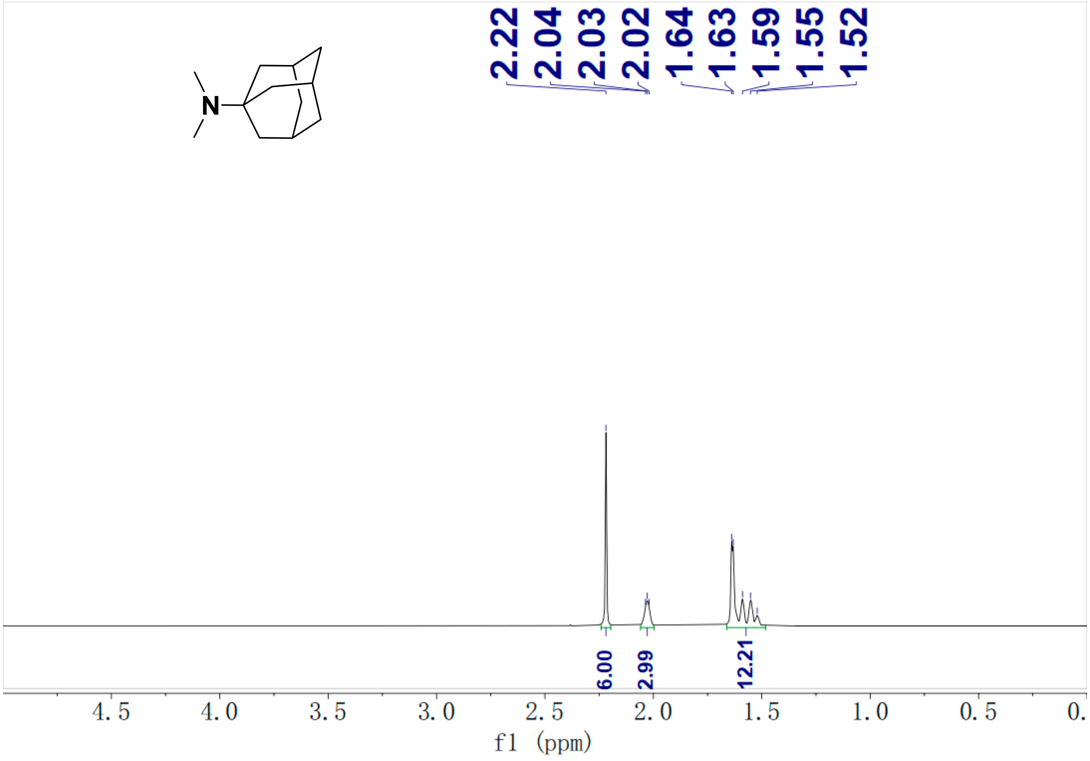

Figure S2. ^{1}H NMR spectrum of dimethyl-1-adamantylamine (2) in $CDCl_3$.

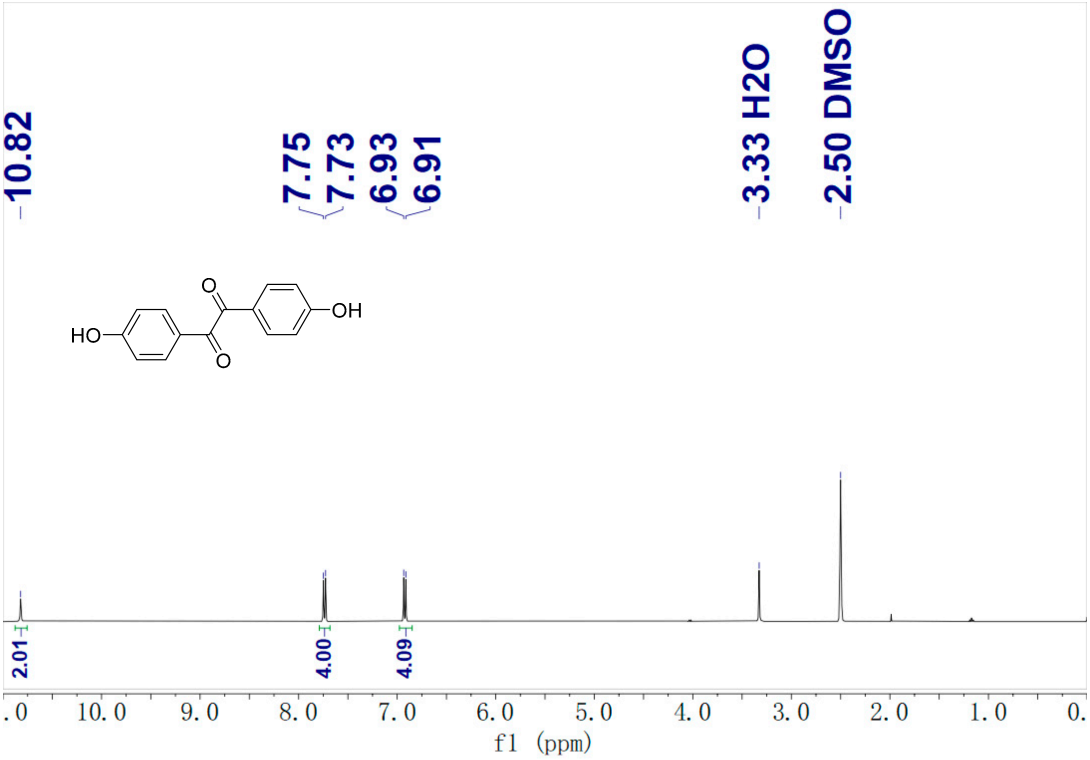

Figure S3. ^{1}H NMR spectrum of 4,4'-dihydroxy-benzil (3) in DMSO.

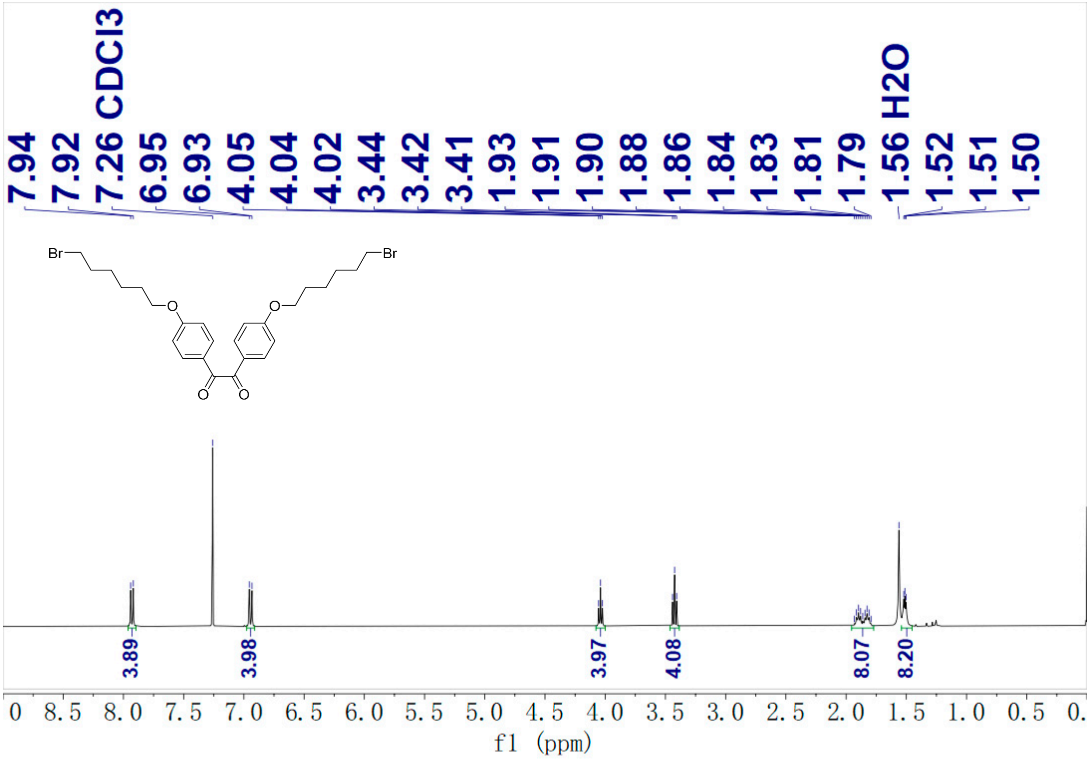

Figure S4. ^{1}H NMR spectrum of compound 4 in DMSO.

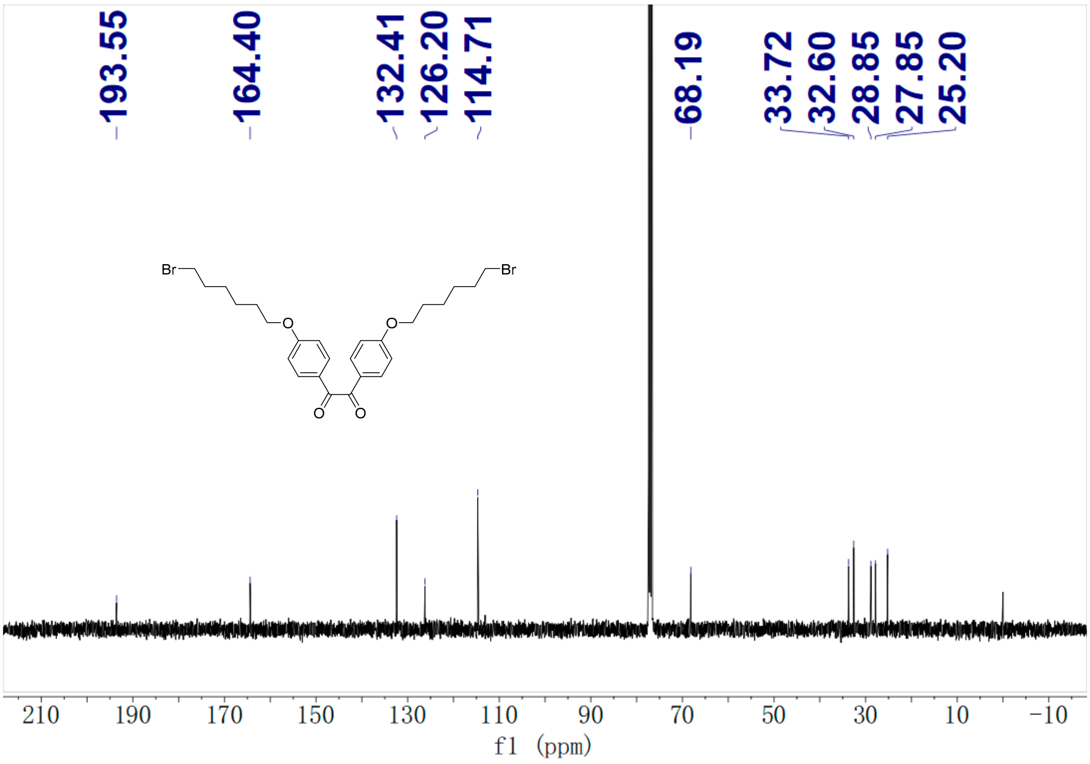

Figure S5. ^{13}C NMR spectrum of compound 4 in DMSO.

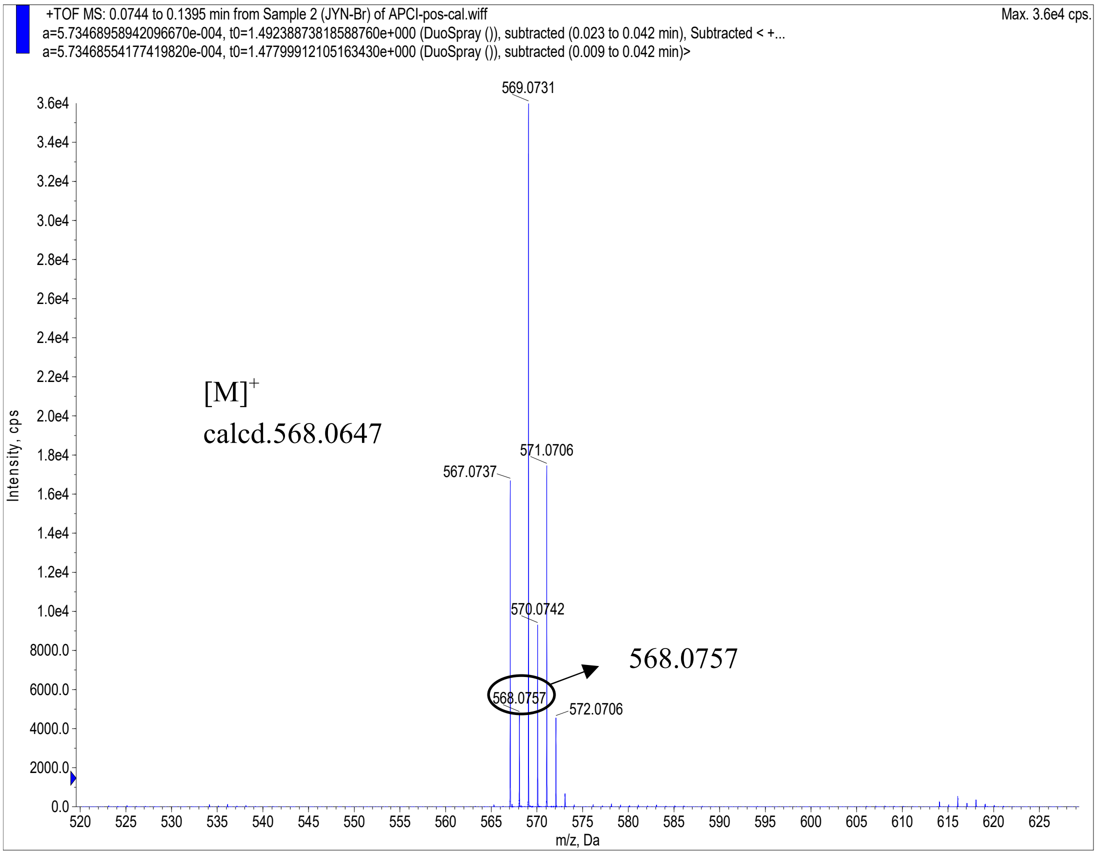

Figure S6. HRMS of Compound 4.

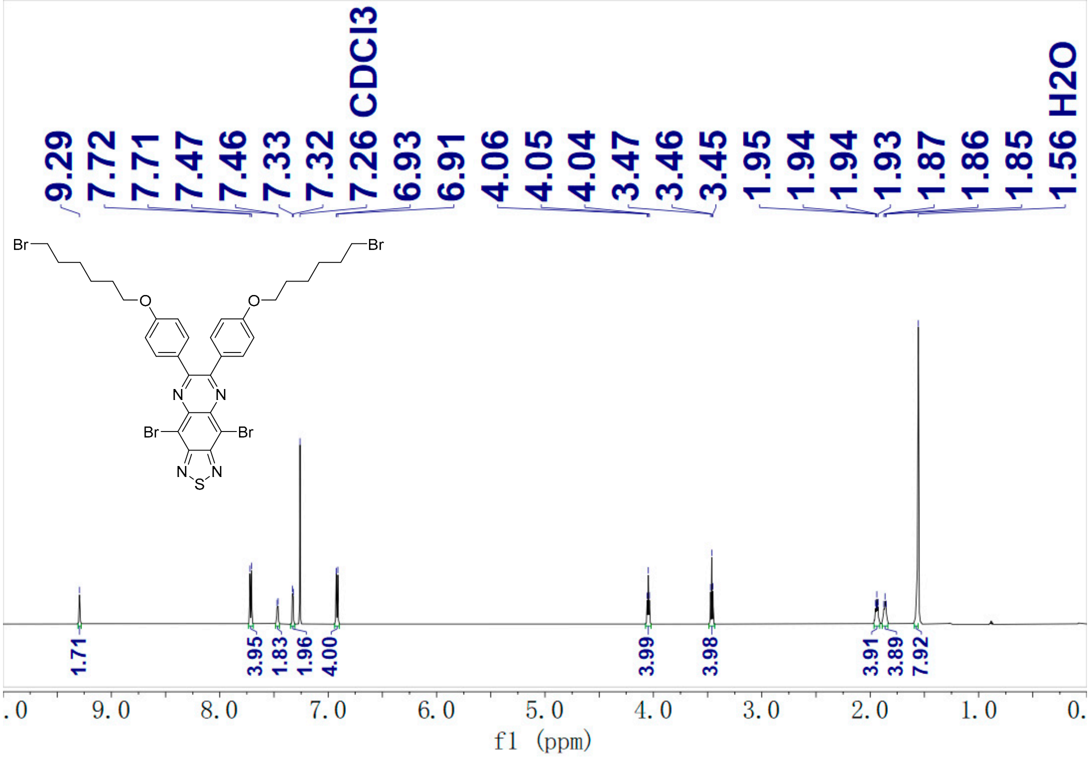

Figure S7. ^{1}H NMR spectrum of compound 5 in $CDCl_3$.

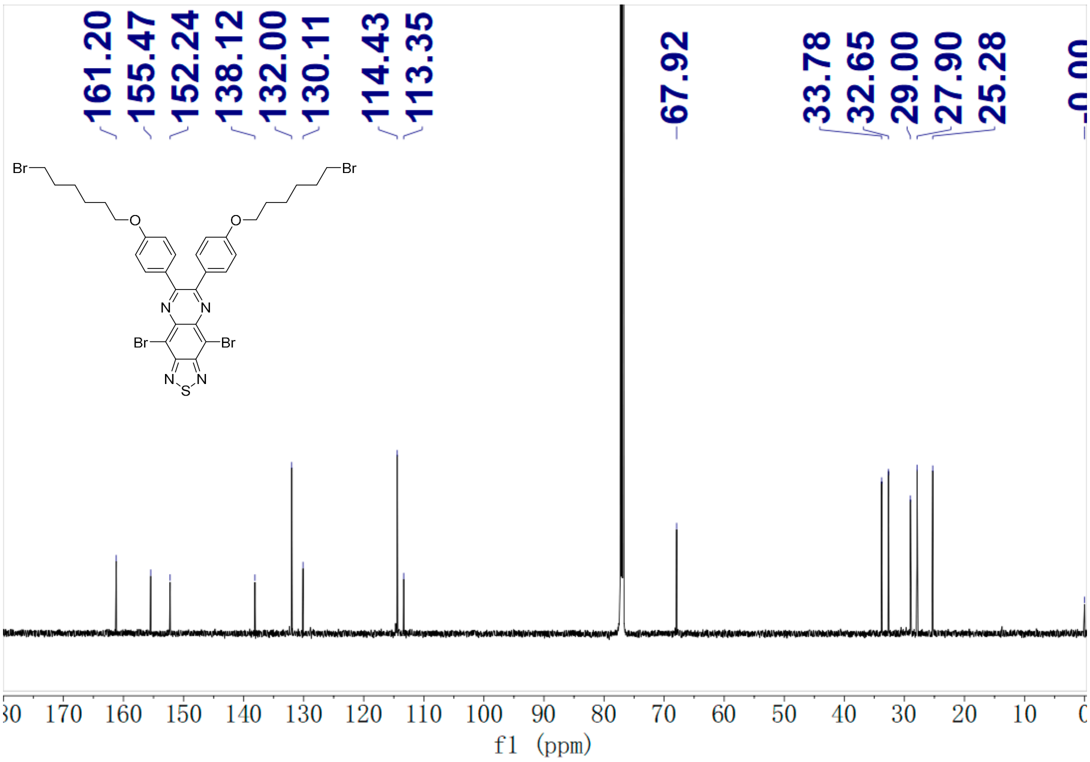

Figure S8. ^{13}C NMR spectrum of compound 5 in $CDCl_3$.

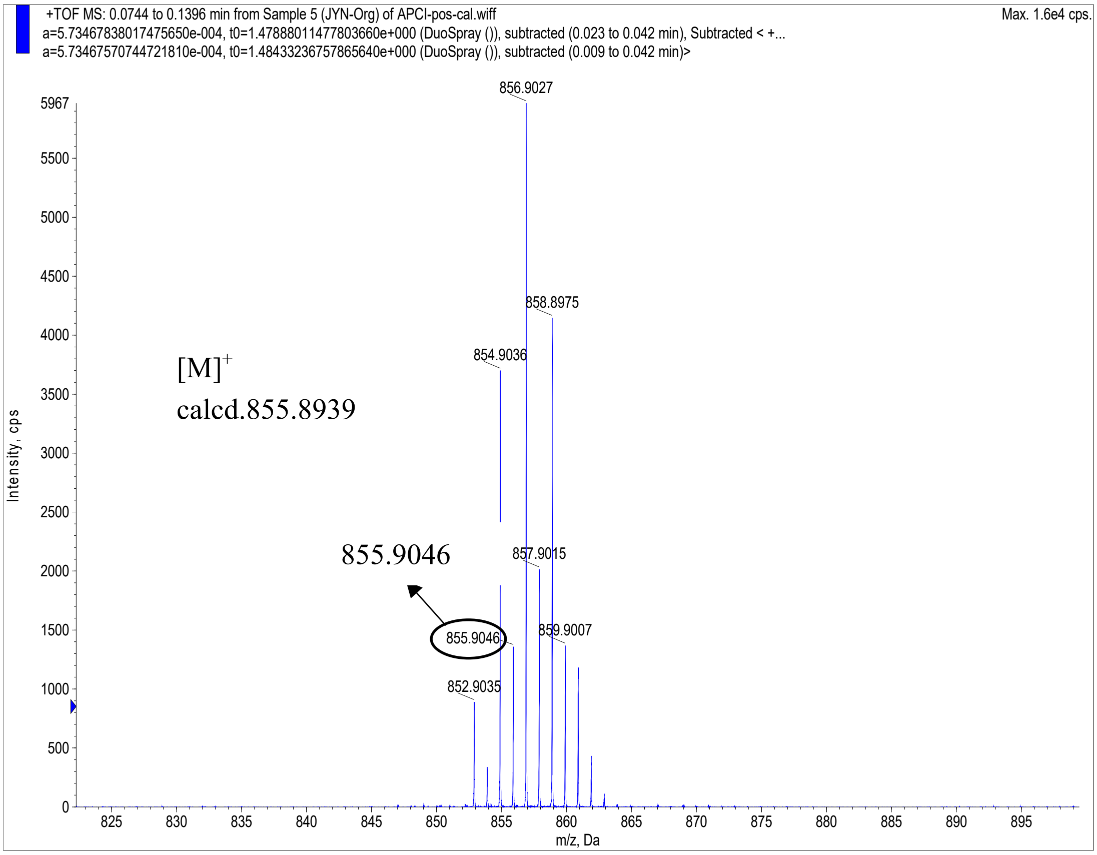

Figure S9. HRMS of compound 5.

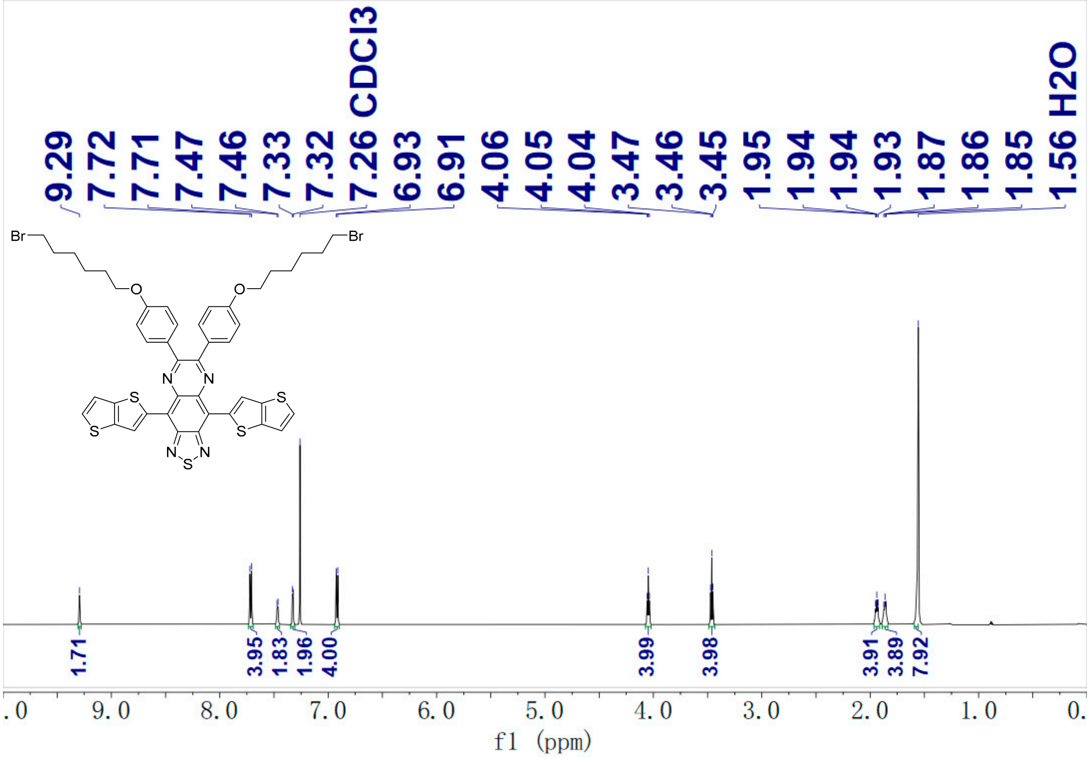

Figure S10. ^{1}H NMR spectrum of compound 6 in $CDCl_3$.

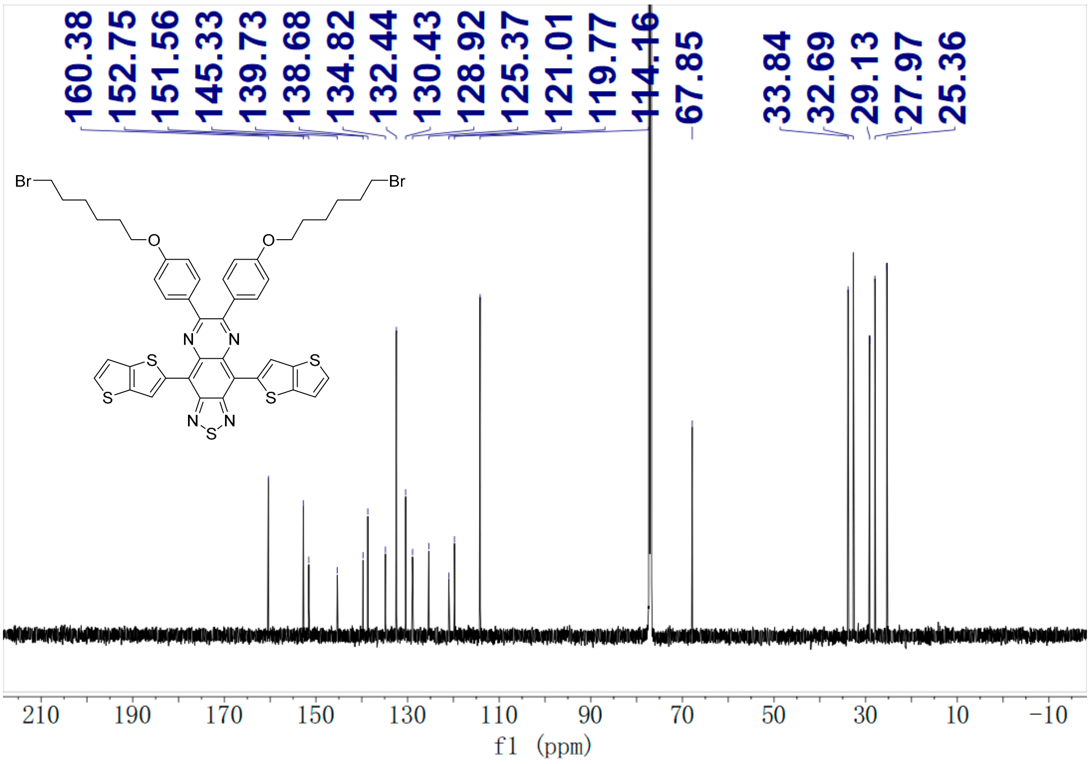

Figure S11. ^{13}C NMR spectrum of compound 6 in $CDCl_3$.

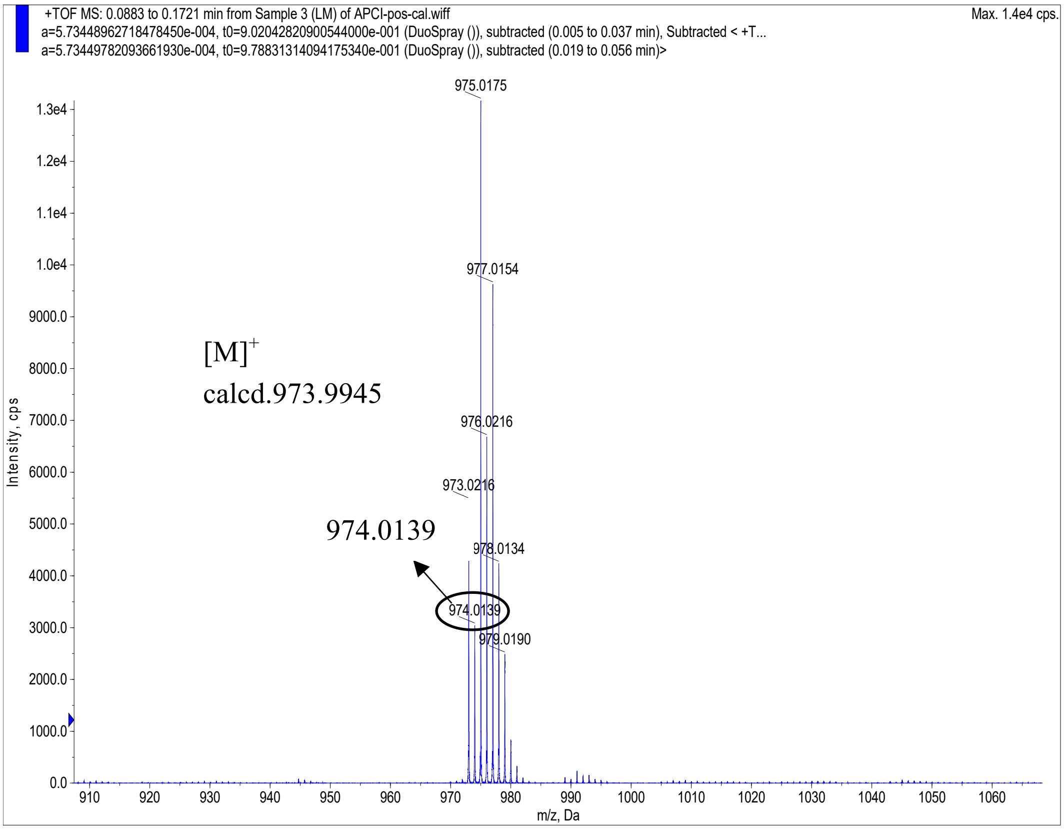

Figure S12. HRMS of compound 6.

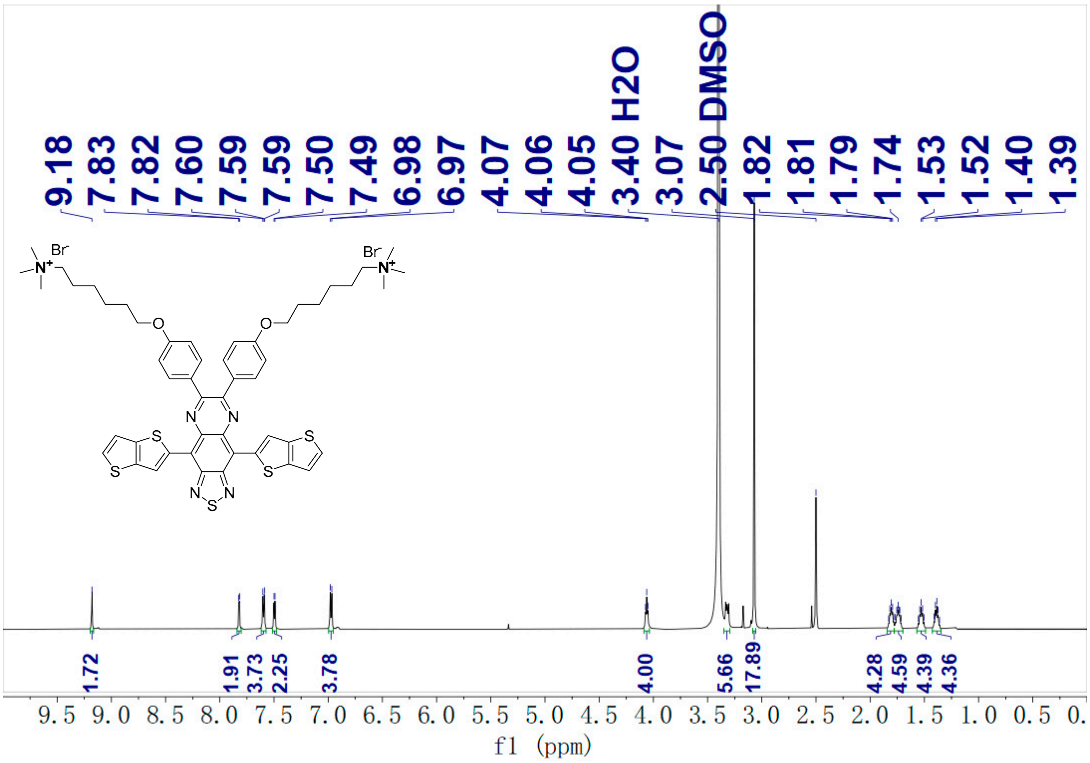

Figure S13. ^{1}H NMR spectrum of TTQTMA in DMSO.

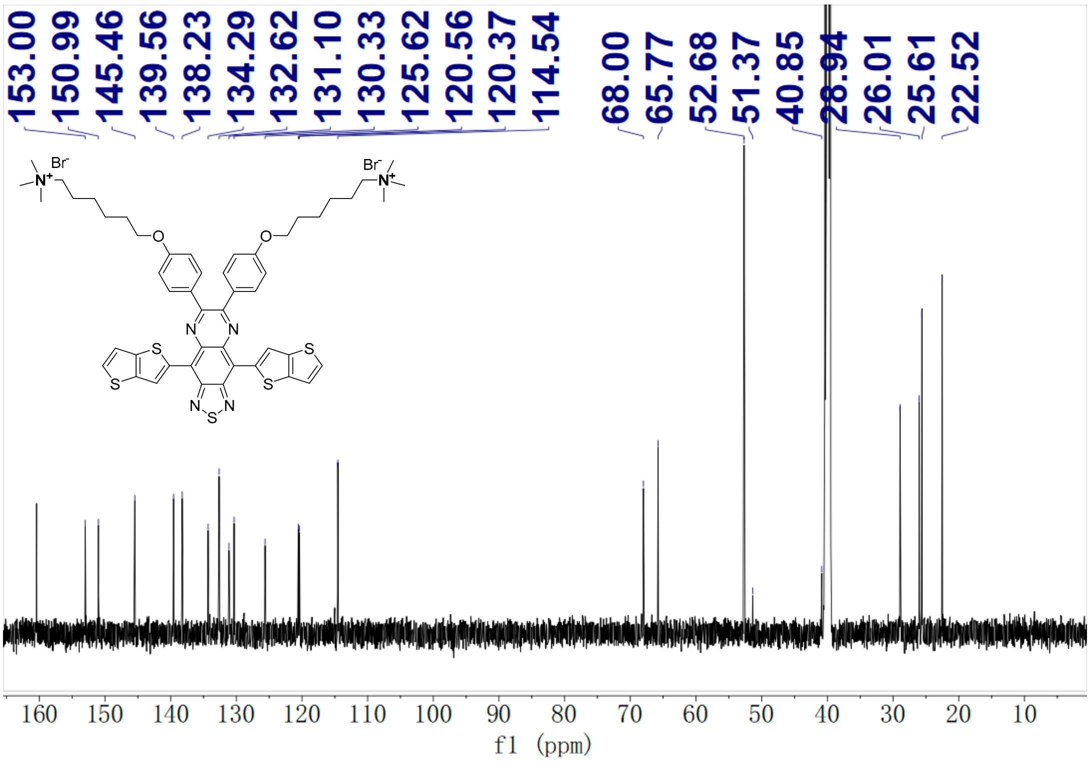

Figure S14. ^{13}C NMR spectrum of TTQTMA in DMSO.

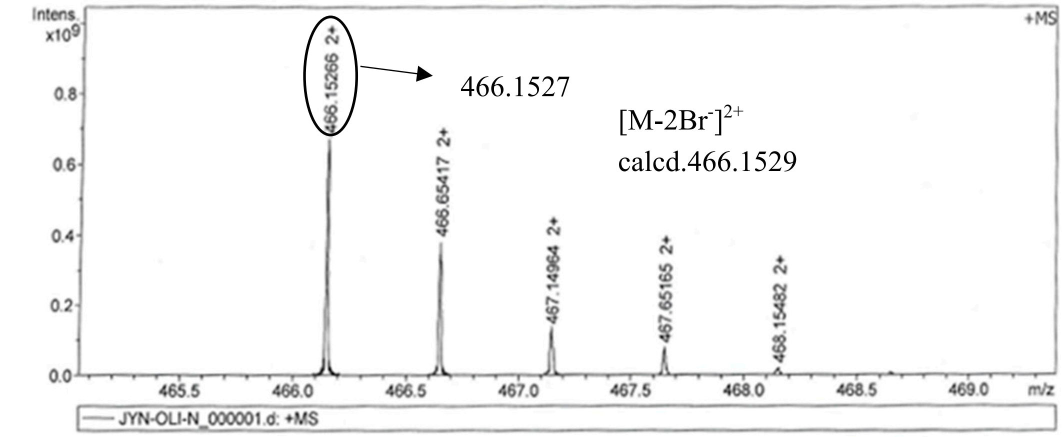

Meas. m/z	#	Ion Formula	Score	m/z	err [ppm]	Mean err [ppm]	mSigma	rdb	e⁻ Conf	N-Rule
466.152662	1	C50H56N6O2S5	100.00	466.152866	0.4	1.2	87.3	26.0	even	ok

Figure S15. HRMS of TTQTMA.

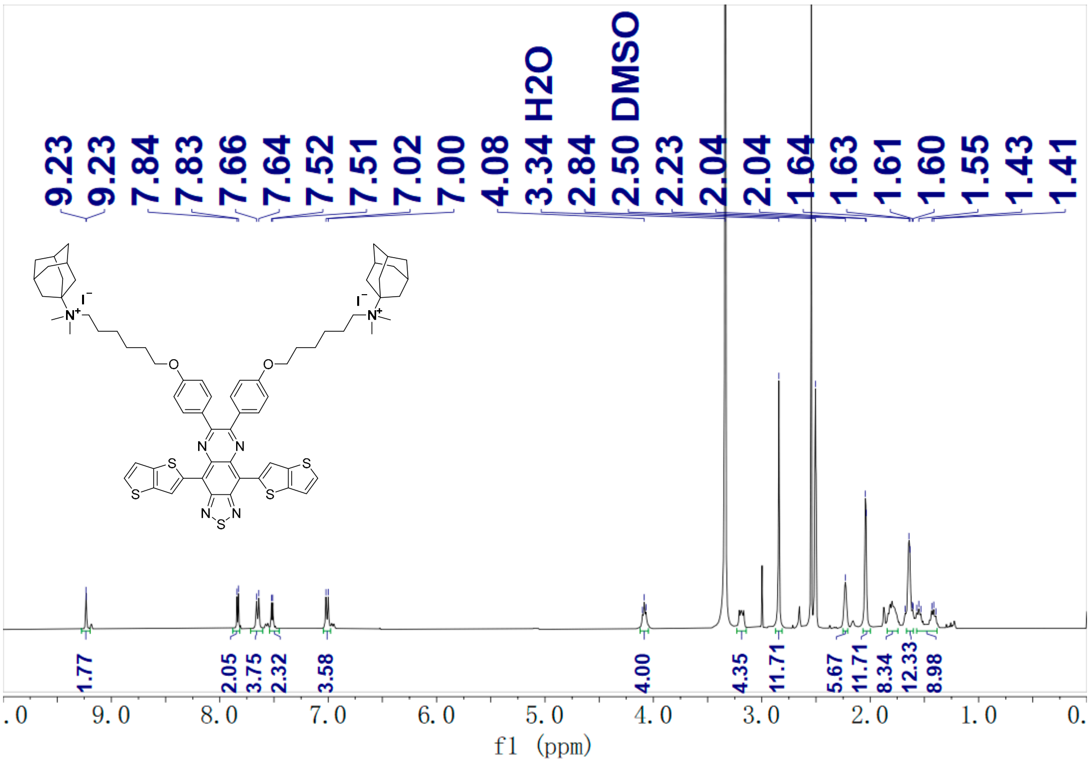

Figure S16. ^{1}H NMR spectrum of TTQAd in DMSO.

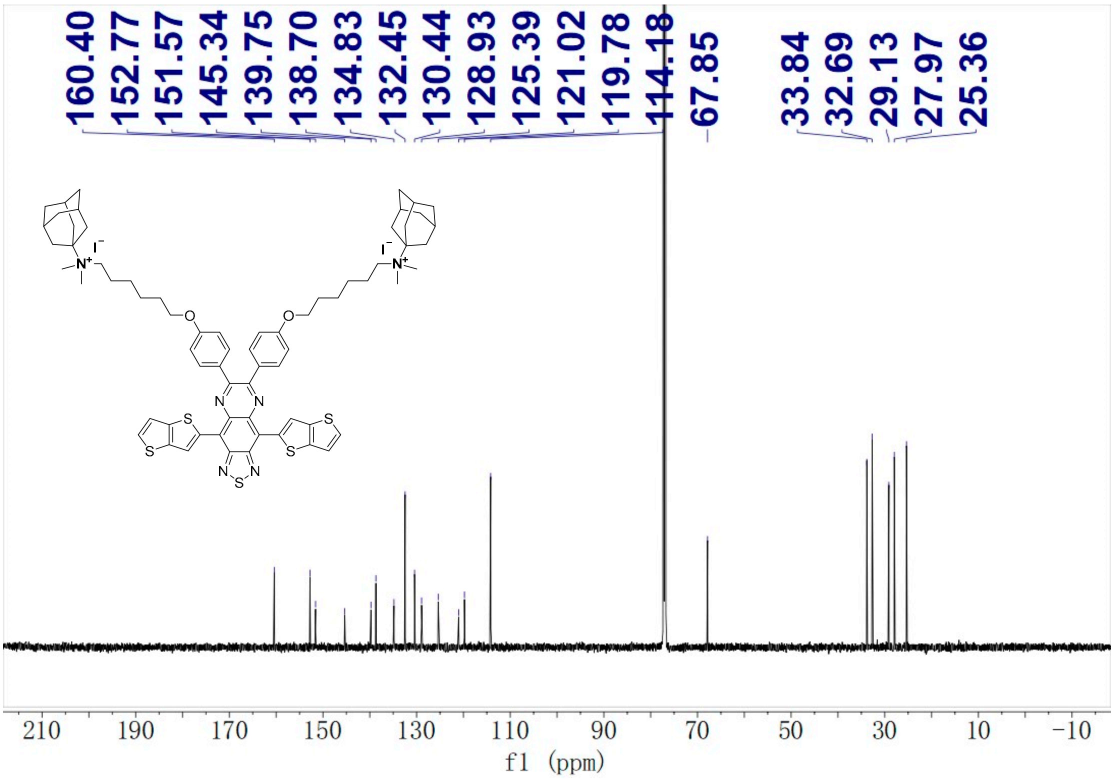

Figure S17. ^{13}C NMR spectrum of TTQAd in DMSO.

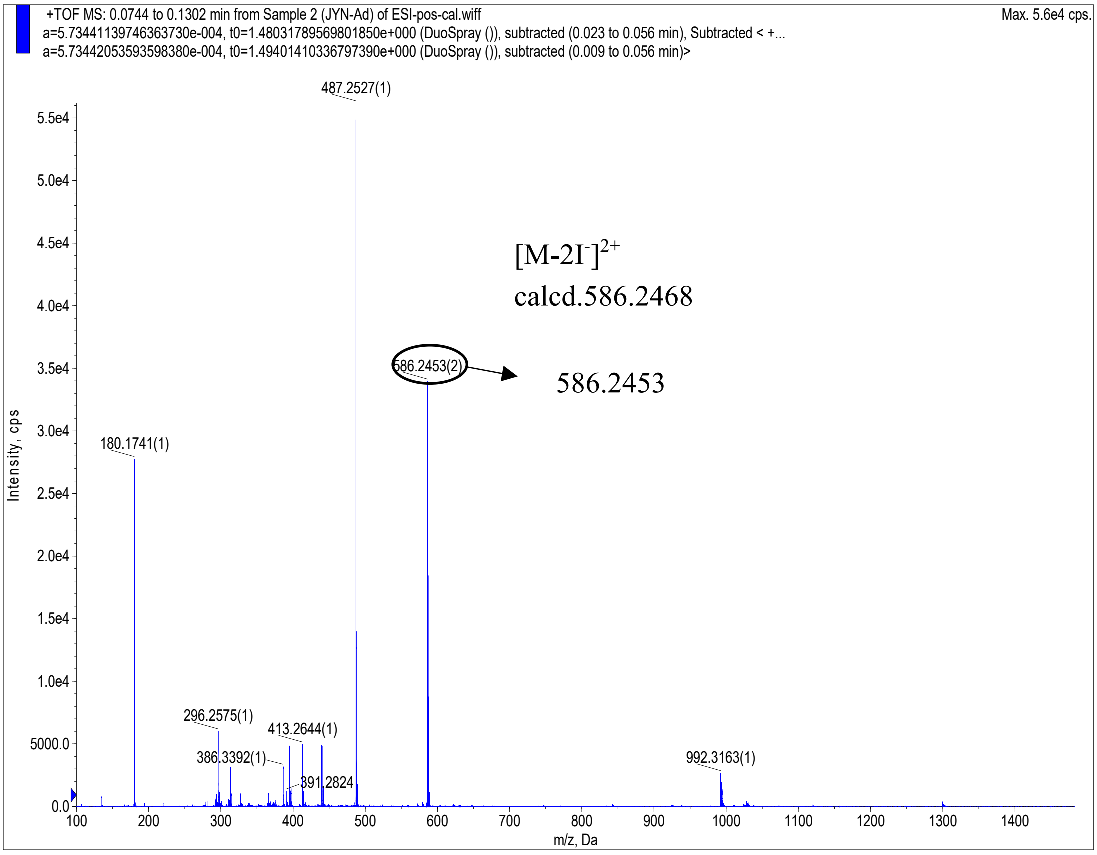

Figure S18. HRMS of TTQAd.

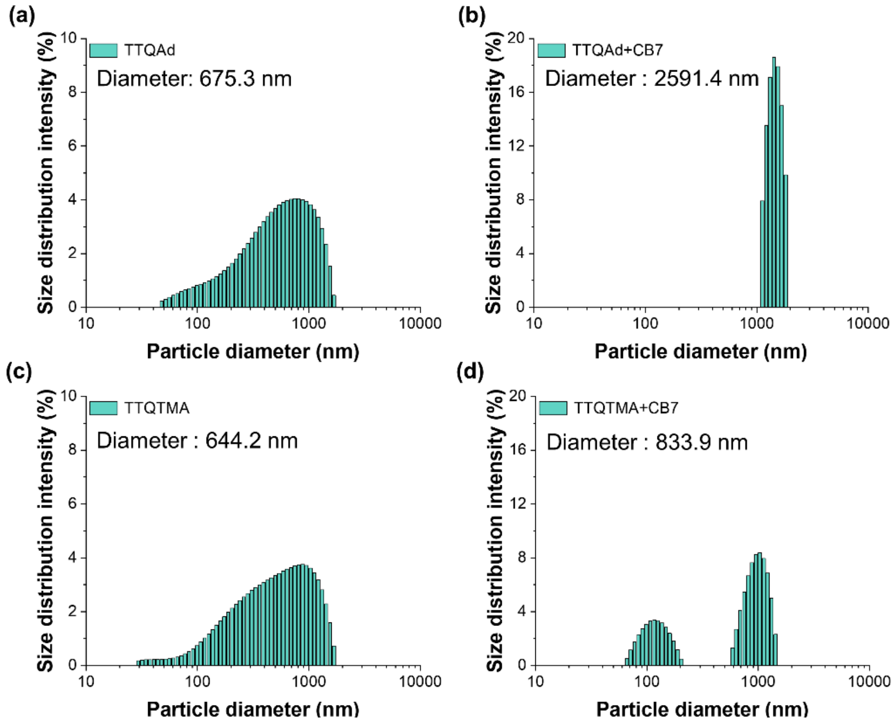

Figure S19. The hydrodynamic diameter histogram of TTQAd and TTQTMA before and after the addition of CB7. (TTQAd/TTQTMA: CB7= 1:2).

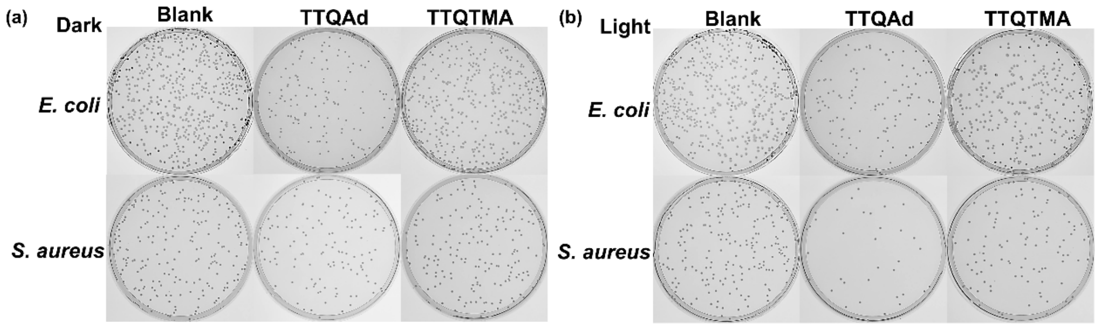

Figure S20. (a) The photographs of plate counting assay of *E. coli* and *S. aureus* treated with TTQAd/TTQTMA (20 μM) in the dark. (b) The photographs of plate counting assay of *E. coli* and *S. aureus* treated with TTQAd/TTQTMA (20 μM) followed by 808 nm laser irradiation (0.5 W cm^{-2}, 10 min).

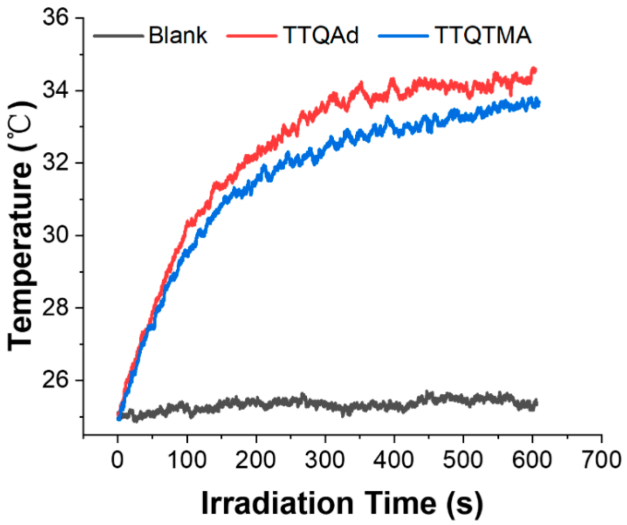

Figure S21. Photothermal conversion of TTQAd and TTQTMA in PBS (20 μM) under 808 nm laser irradiation (0.5 W cm^{-2}, 10 min).

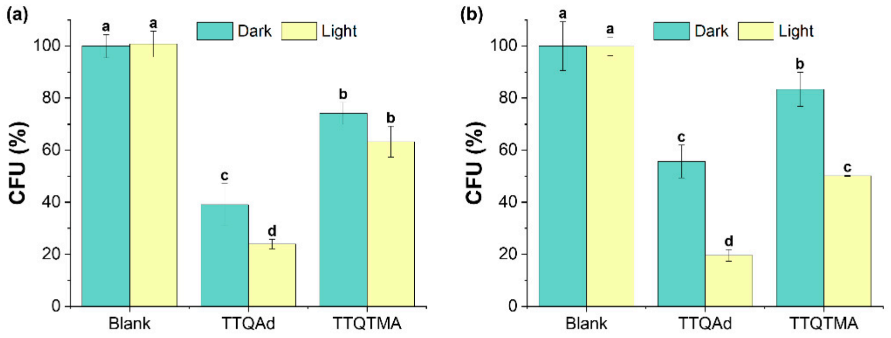

Figure S22. Bacterial viabilities of *E. coli* (a) and *S. aureus* (b) treated with TTQAd/TTQTMA (20 μM) with/without 808 nm laser irradiation (0.5 W cm^{-2}, 10 min). Different letters (a, b, c, d) in the bar graphs represent significant difference at $p < 0.05$ levels.

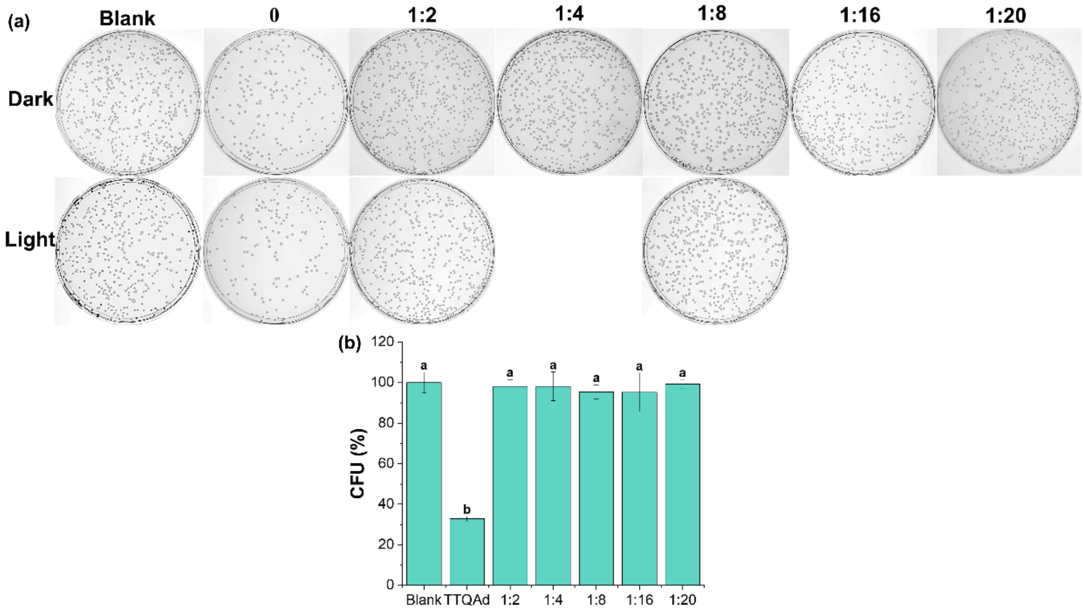

Figure S23. Photographs of *E. coli* colonies on agar plates (a) and bacterial viabilities of *E. coli* (b) treated with TTQAd before and after the addition of different amounts of CB7 with/without 808 nm laser irradiation (0.5 W cm^{-2}). [TTQAd] = 20 μM, [CB7] = 0, 40, 80, 160, 320, 400 μM. Different letters (a, b, c, d) represent significant difference at $p < 0.05$ levels.

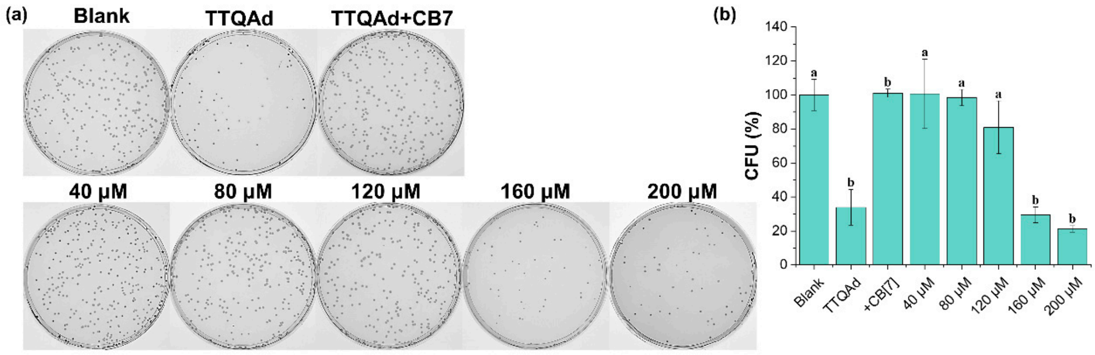

Figure S24. Photographs of *E. coli* colonies on agar plates (a) and bacterial viabilities of *E. coli* (b) treated with TTQAd-CB7 and different amounts of TMeAd. [TTQAd-CB7] = 20 μM, [TMeAd] = 0, 40, 80, 120, 160, 200 μM. Different letters (a, b) in the bar graph represent significant difference at $p < 0.05$ levels.

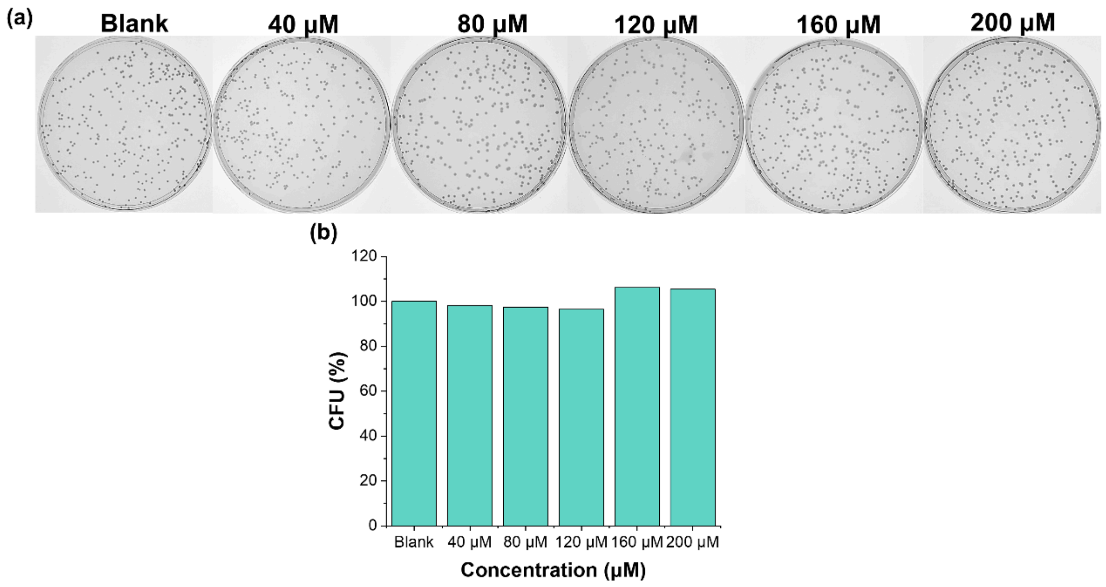

Figure S25. Photographs of *E. coli* colonies on agar plates (a) and bacterial viabilities of *E. coli* (b) treated with TMeAd. [TMeAd] = 0, 40, 80, 120, 160, 200 μM.

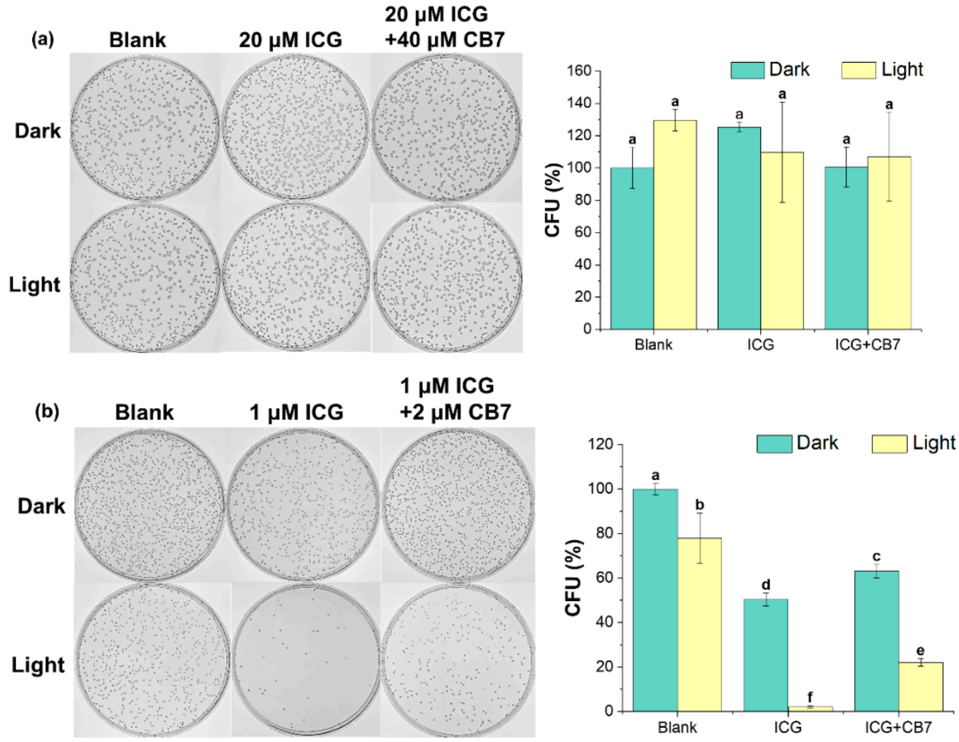

Figure S26. (a) Photographs of *E. coli* colonies on agar plates and bacterial viabilities of *E. coli* treated with ICG (20 μM) before and after the addition of CB7 (40 μM) with/without 808 nm laser irradiation (0.5 W cm^{-2}, 10 min); (b) Photographs of *S. aureus* colonies on agar plates and bacterial viabilities of *S. aureus* treated with ICG (1

μM) before and after the addition of CB7 (2 μM) with/without 808 nm laser irradiation (0.5 W cm^{-2}, 10 min); Different letters (a, b, c, d) in the bar graphs represent significant difference at $p < 0.05$ levels.

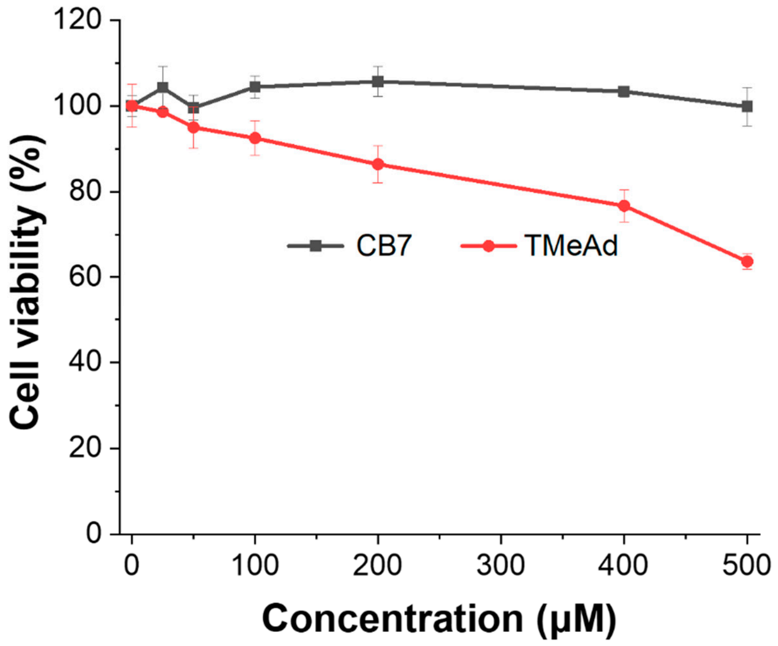

Figure S27. Cell viability after treating with CB7 and TMeAd at different concentrations in the absence of light.
